# The circular RNA circP5CS1 coordinates plant immunity by sequestering miR167 to modulate ARF6/8 signaling and repressing P5CS1-mediated proline biosynthesis in *Arabidopsis*

**DOI:** 10.1016/j.xplc.2026.101922

**Published:** 2026-05-20

**Authors:** Lin Wang, Xiaoning Song, Ran Wei, Xuechao Shi, Le Xu, Ze Yu, Cheng Tang, Leyao Li, Baohuan Guo, Hongxia Liu, Xiaoyong Sun, Hongwei Zhao

**Affiliations:** 1 College of Life and Environmental Science, Wenzhou University, Wenzhou 325035, China; 2 State Key Laboratory of Agricultural and Forestry Biosecurity, College of Plant Protection, Nanjing Agricultural University, Nanjing 210095, China; 3 Key Laboratory of Integrated Management of Crop Disease and Pests, Ministry of Education, Nanjing Agricultural University, Nanjing 210095, China; 4 Agricultural Big-Data Research Center, College of Information Science and Engineering, Shandong Agricultural University, Taian 271018, China

**Keywords:** circular RNA, miR167, *P5CS1*, proline metabolism, *Arabidopsis thaliana*

## Abstract

Circular RNAs (circRNAs) are a type of noncoding RNA formed by backsplicing, characterized by covalently closed loop structures with enhanced stability. Although growing evidence highlights their regulatory roles in plants, their precise biological functions remain largely unclear. In this study, we identified *Arabidopsis thaliana* circP5CS1, a circRNA derived from the delta-1-pyrroline-5-carboxylate synthase (*P5CS1*) gene through backsplicing of exons 11–13. The junction between exons 11 and 13 generates a *de novo* miR167 binding site, confirmed by sequence alignment and RNA pull-down assays, indicating that circP5CS1 preferentially binds miR167a/b. circP5CS1 acts as a miR167 sponge to promote the expression of auxin response factor 6 (*ARF6*) and *ARF8*, fine-tuning salicylic acid- and jasmonic acid-mediated immunity during infection by *Pseudomonas syringae* pv. tomato DC3000. In addition, circP5CS1 suppresses its host gene *P5CS1*, a rate-limiting enzyme for proline biosynthesis, through both splicing competition and post-transcriptional mechanisms, thus linking circRNA biogenesis with the control of gene expression. Our results suggest that circP5CS1 directly modulates plant immunity through both hormone and proline pathways. circP5CS1 expression can be induced by both *P. syringae pv. tomato* DC3000 and *AvrRpt2* infection. Accordingly, resistance to these pathogens is compromised in circP5CS1-overexpression (OE), *p5cs1*, and miR167 short tandem target mimic plants, whereas circP5CS1-RNAi, P5CS1-1-OE, and miR167-OE plants show enhanced resistance. Proline levels are increased in P5CS1-1-OE and circP5CS1-RNAi plants but reduced in circP5CS1-OE and *p5cs1* mutants, supporting a link between circP5CS1 activity and proline metabolism. Our findings suggest that circP5CS1 may be an immune regulatory hub that integrates hormone signaling and proline homeostasis through microRNA sponging and host-gene suppression, revealing its dual regulatory role in coordinating RNA-mediated regulation with plant immune response.

## Teaser

This study demonstrates that circP5CS1 functions as a regulatory hub linking miR167 signaling, proline metabolism, and immunity in *Arabidopsis*. Mechanistically, circP5CS1 sequesters miR167a/b to modulate ARF6/8 signaling while repressing the expression of its host gene, *P5CS1*, thereby coordinating hormone-associated defense responses and proline biosynthesis during pathogen infection.

## Introduction

Plants constantly face pathogen threats that require precise coordination of immune activation and growth regulation (Gonzalez et al., 2015; Delplace et al., 2022; He et al., 2022). This balance is particularly critical during biotic stress, when transcriptional programs must rapidly integrate hormonal signaling and metabolic adaptation to mount effective defenses (Saijo and Loo, 2020; Varadharajan et al., 2025). Among post-transcriptional regulators, microRNAs (miRNAs) play a central role in orchestrating stress-responsive gene expression (Covarrubias and Reyes, 2010). miR167 regulates *ARF6* and *ARF8* expression, thereby modulating the auxin-mediated pathways that influence stomatal behavior, cell expansion, and immunity (Ru et al., 2006; Wu et al., 2006; Su et al., 2016; Caruana et al., 2020). Recent studies have highlighted a critical role for miR167 in plant immunity in addition to its regulation of auxin-driven developmental processes. In *Arabidopsis*, overexpression (OE) of miR167 confers enhanced resistance to *Pseudomonas syringae* by activating salicylic acid (SA)-dependent defense pathways, whereas miR167 suppression compromises basal immunity and alters auxin–SA crosstalk (Caruana et al., 2020). In rice, Osa-miR167d promotes *Magnaporthe oryzae* infection by downregulating *OsARF12*, increasing host susceptibility (Zhao et al., 2020). Evidence from cereals and solanaceous species provides further evidence that *ARF*-targeting miR167 (Zhao et al., 2020) is a conserved regulator that coordinates auxin and defense signaling (Baldrich and San Segundo, 2016). These results establish circP5CS1 as a pivotal regulatory hub that integrates hormone signaling with defense responses. Although the downstream functions of miR167 are well established, how its abundance and activity are dynamically regulated during pathogen infection remains unclear. Recent discoveries have identified circular RNAs (circRNAs), a class of covalently closed, noncoding RNAs, as emerging players in gene regulation through miRNA sequestration. However, their functional relevance in shaping immune-responsive networks in plants has yet to be fully explored.

circRNAs are a unique class of noncoding RNAs generated through backsplicing that form covalently closed loop structures with high stability (Cheng et al., 2018; Chen, 2020). In animal systems, circRNAs have been extensively characterized as miRNA sponges that modulate post-transcriptional regulation (Hansen et al., 2013; Memczak et al., 2013; Panda, 2018). More recently, functional studies of circRNAs have been reported in plants, revealing a broader spectrum of regulatory roles (Zhang et al., 2024). In addition to acting as miRNA sponges, plant circRNAs have been shown to modulate the expression of their linear host genes, influencing transcription, splicing, and stress-responsive pathways (Fan et al., 2020; Zhang and Dai, 2022; Liu et al., 2023, 2025; Pan et al., 2025; Tang et al., 2025). Although several plant circRNAs have been experimentally linked to stress responses, the mechanisms that underlie their involvement in immunity, particularly their integration into miRNA and host-gene regulatory circuits, are incompletely understood.

In our previous transcriptomic analysis, we identified a set of circRNAs in *Arabidopsis thaliana* whose expression appeared to be responsive to pathogen challenge (Sun et al., 2016; Wang et al., 2022). One candidate, circR5998 (Wang et al., 2022), hereafter referred to as circP5CS1, is derived from the *P5CS1* locus (*AT2G39800*), a gene involved in proline biosynthesis (Mattioli et al., 2008). Proline accumulation is tightly linked to stress adaptation through its roles in osmotic balance, redox buffering, and immune modulation (Liang et al., 2013; Qamar et al., 2015; Alvarez et al., 2022; Ghosh et al., 2022). As well as having a classic function in abiotic stress responses, proline metabolism has emerged as a central player in plant immunity. In *Arabidopsis*, proline dehydrogenase 1 (PRODH1), which catalyzes the first step of proline catabolism, is strongly induced upon perception of flagellin (flg22) and promotes pattern-triggered immunity by enhancing respiratory burst oxidase homolog-mediated reactive oxygen species (ROS) production and activating mitogen-activated protein kinase cascades (Fabro et al., 2016). Loss-of-function *prodh1* mutants exhibit impaired ROS bursts, diminished expression of immune marker genes, and increased susceptibility to bacterial infection. Furthermore, the catabolic intermediate delta-1-pyrroline-5-carboxylate (P5C) functions as a redox-active signaling molecule that facilitates hypersensitive response (HR)-associated cell death during effector-triggered immunity (Cecchini et al., 2011). These findings reveal that proline homeostasis, particularly the Pro–P5C cycle, is intimately linked to immune signal transduction. However, it remains unclear how *P5CS1* expression is modulated at the post-transcriptional level during pathogen attack and whether noncoding RNA-based regulatory mechanisms have a role in immunometabolic coordination.

circP5CS1 was predicted to be upregulated in response to bacterial infection and was confirmed as a *bona fide* circRNA through junction-specific assays (Sun et al., 2016). Given its induced expression and the presence of putative miR167-binding sites, we hypothesized that circP5CS1 might function as a decoy for miR167 target genes, modulating gene networks associated with auxin signaling and proline biosynthesis during pathogen stress. To investigate the functional role of circP5CS1 in pathogen-induced responses, we combined transgenic analysis, RNA interaction assays, and immune phenotyping in *A. thaliana*. Our results demonstrate that circP5CS1 not only acts as a molecular decoy for *ARF6* and *ARF8*, modulating the hormone signaling associated with plant defense responses, but also affects the expression of its host gene, *P5CS1*, which is critical for osmotic balance, redox buffering, and immune modulation. Its effects on proline accumulation and hormone signaling have a significant influence on plant defense against *P. syringae pv. tomato* DC3000 and its effector strain *AvrRpt2*, which elicit basal and effector-triggered defense, respectively. Although these findings reveal a specific post-transcriptional circuit involving circRNA–miRNA crosstalk, how such mechanisms broadly integrate hormonal and metabolic pathways in plant immunity has been unclear. Addressing this knowledge gap, our study reveals a circRNA-centered regulatory module that links auxin signaling and proline metabolism, offering new insight into the coordination of growth and defense at the RNA level.

## Results

### Molecular characterization and expression engineering of circP5CS1

circP5CS1 is derived from exons 11–13 of the *P5CS1* gene, a sequence preserved across all four transcript isoforms (Figure 1A). As in our earlier report, we validated circP5CS1 through RNase R digestion and reverse-transcription polymerase chain reaction (RT–PCR) with divergent primers, complemented by poly(A)-based RNA fractionation to confirm its circular structure and lack of polyadenylation (Wang et al., 2022).

**Figure 1:**
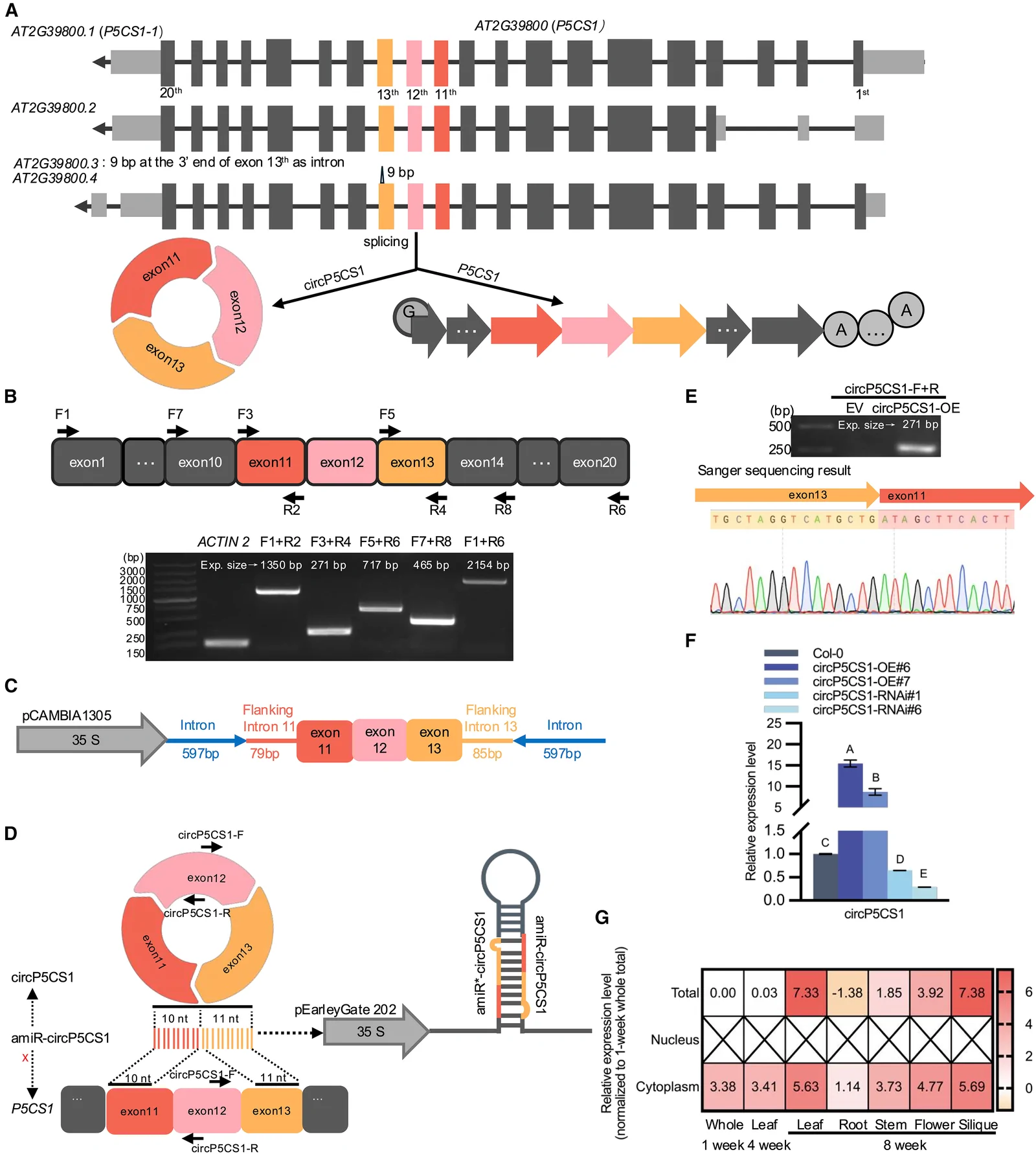
Transcript validation and genetic tools for specific regulation of circP5CS1 in *Arabidopsis*. **(A)** A schematic of the *P5CS1* (*AT2G39800*) gene, showing four annotated transcript isoforms. circP5CS1 emerges from exons 11–13 of *P5CS1*, a sequence preserved across all isoforms. **(B)** Top: RT–PCR analysis of P5CS1 transcripts in Col-0 is depicted by boxes (exons) and arrows (PCR primers). Exon-spanning primer pairs were used: F1+R2 (exons 1–11), F3+R4 (exons 11–13), F5+R6 (exons 13–20), F7+R8 (exons 10–14), and F1+R6 (exons 1–20). *Arabidopsis ACTIN 2* served as the control gene. Bottom: gel electrophoresis of amplified bands using exon-spanning primers. Expected sizes are labeled over the corresponding bands. **(C)** A schematic of the circP5CS1-OE construct, including exons 11–13 and their flanking introns, together with two 597-bp reverse complementary sequences derived from *AT2G39720* intron 1, designed to facilitate circularization. The expression cassette was driven by the CaMV 35S promoter. **(D)** Design of a circP5CS1-RNAi construct expressing an amiRNA that targets the backsplice junction of circP5CS1. This approach enables selective knockdown of the circular transcript, leaving the linear P5CS1 mRNA unaffected. **(E)** Top: RT–PCR detection of circP5CS1 in *N. benthamiana* leaves transiently expressing the circP5CS1-OE construct. Divergent primers spanning the backsplice junction were used to amplify circP5CS1. The empty vector was used as a control. Bottom: Sanger sequencing of the RT–PCR product confirmed the precise head-to-tail back-splicing of exons 11–13. **(F)** RT–qPCR quantification of circP5CS1 expression in Col-0, OE, and RNAi transgenic lines using divergent primers specific to the backsplice junction. Significant upregulation of circP5CS1 is observed in OE lines, in contrast to downregulation in RNAi lines. The data are presented as mean ± SEM (*n* = 3 biological replicates), with A–E denoting significance (*p* < 0.01, one-way ANOVA followed by Duncan’s test for multiple comparisons). **(G)** Subcellular localization and tissue-specific accumulation of circP5CS1. RNA was isolated from total, nuclear, and cytoplasmic fractions of 1-week-old whole seedlings, 4-week-old leaves, and various 8-week-old tissues (leaves, roots, stems, flowers, and siliques). circP5CS1 levels were quantified by RT–qPCR and visualized as a heatmap, normalized to total RNA from 1-week-old seedlings. The data are presented as mean ± SEM from 3 biological replicates (*n* = 3).

To determine whether circP5CS1 biogenesis (the head-to-tail circularization of exons 11–13) interferes with canonical splicing of the host transcript, we examined the integrity and expression level of 5 *P5CS1* transcription fragments (exons 1–11, 11–13, 13–20, 10–14, and 1–20) by RT–PCR across all 4 annotated *P5CS1* isoforms (Figure 1B), focusing on the detection of potential alternative splicing events, particularly exon skipping. The results revealed neither aberrant splice variants nor exon skipping around the circularization site (Figure 1B), suggesting that the formation of circP5CS1 does not interfere with the canonical splicing pattern of *P5CS1* under physiological conditions.

To enable targeted modulation of circP5CS1, we generated transgenic *Arabidopsis* lines with altered expression. For OE, exons 11–13 and their original up- and downstream introns (introns 11 and 13) were cloned as the core circularization cassette, driven by a 35S promoter. To increase circularization efficiency (Zhang et al., 2014; Gao et al., 2019), a pair of reverse complementary intronic fragments derived from the first intron of *AT2G39720* was incorporated, as this intron is relatively long and was selected without functional bias (Figure 1C). For knockdown, an artificial miRNA (amiRNA) was constructed to specifically target the backsplice junction of circP5CS1 while avoiding matching the linear transcript (Figure 1D). To verify that the circP5CS1-OE construct enabled precise circularization of circP5CS1 *in planta*, we performed *Agrobacterium*-mediated transient expression in *Nicotiana benthamiana*. Leaf tissues were collected 2 days post-infiltration for total RNA extraction. After reverse transcription, RT–PCR was performed using divergent circP5CS1-specific primers to amplify across the backsplice junction. Gel electrophoresis confirmed the presence of circP5CS1-specific bands in the OE group but not in the empty-vector negative control (Figure 1E, top). The amplified products were further validated by Sanger sequencing, which confirmed precise back-splicing and accurate circular junction formation (Figure 1E, bottom). These results demonstrate that the circP5CS1-OE vector efficiently directs the generation of authentic circP5CS1 molecules *in planta*. Quantitative RT–PCR (RT–qPCR) using junction-specific primers confirmed substantial upregulation of circP5CS1 in OE lines and marked suppression in RNAi lines relative to Col-0 controls (Figure 1F).

To investigate the subcellular localization of circP5CS1, we performed RNA fractionation of different tissues and developmental stages, followed by RT–qPCR. circP5CS1 was undetectable in the nuclear fraction and predominantly localized in the cytoplasm across all tested samples (Figure 1G). Notably, its cytoplasmic accumulation was higher in 8-week-old leaves and siliques than in 4-week-old leaves and 1-week-old seedlings. Levels of circP5CS1 generally increased with plant age, with roots showing relatively low expression. These results suggest that circP5CS1 is primarily cytoplasmic and exhibits tissue- and age-dependent accumulation, supporting its role in post-transcriptional regulation.

### Reciprocal regulation between circP5CS1 and *P5CS1* transcript levels in *Arabidopsis*

To investigate the potential regulatory relationship between circP5CS1 and its host gene *P5CS1*, we reanalyzed publicly available RNA sequencing datasets from 17 *Arabidopsis* mutants impaired in RNA processing or splicing (Philips et al., 2020). P5CS1 transcript abundance was reduced in most mutants relative to Col-0, with the exception of *rdr4* (Figure 2A, top), which showed a modest increase. Isoforms 2 and 3 were the most abundant *P5CS1* isoforms in wild-type *Arabidopsis*, but isoforms 1 and 2 consistently accounted for the majority of *P5CS1* expression in the RNA-processing mutants, and levels of isoforms 3 and 4 were low across most backgrounds (Figure 2A, bottom). These results suggest that splicing-related factors modulate *P5CS1* expression in an isoform-dependent manner.

**Figure 2:**
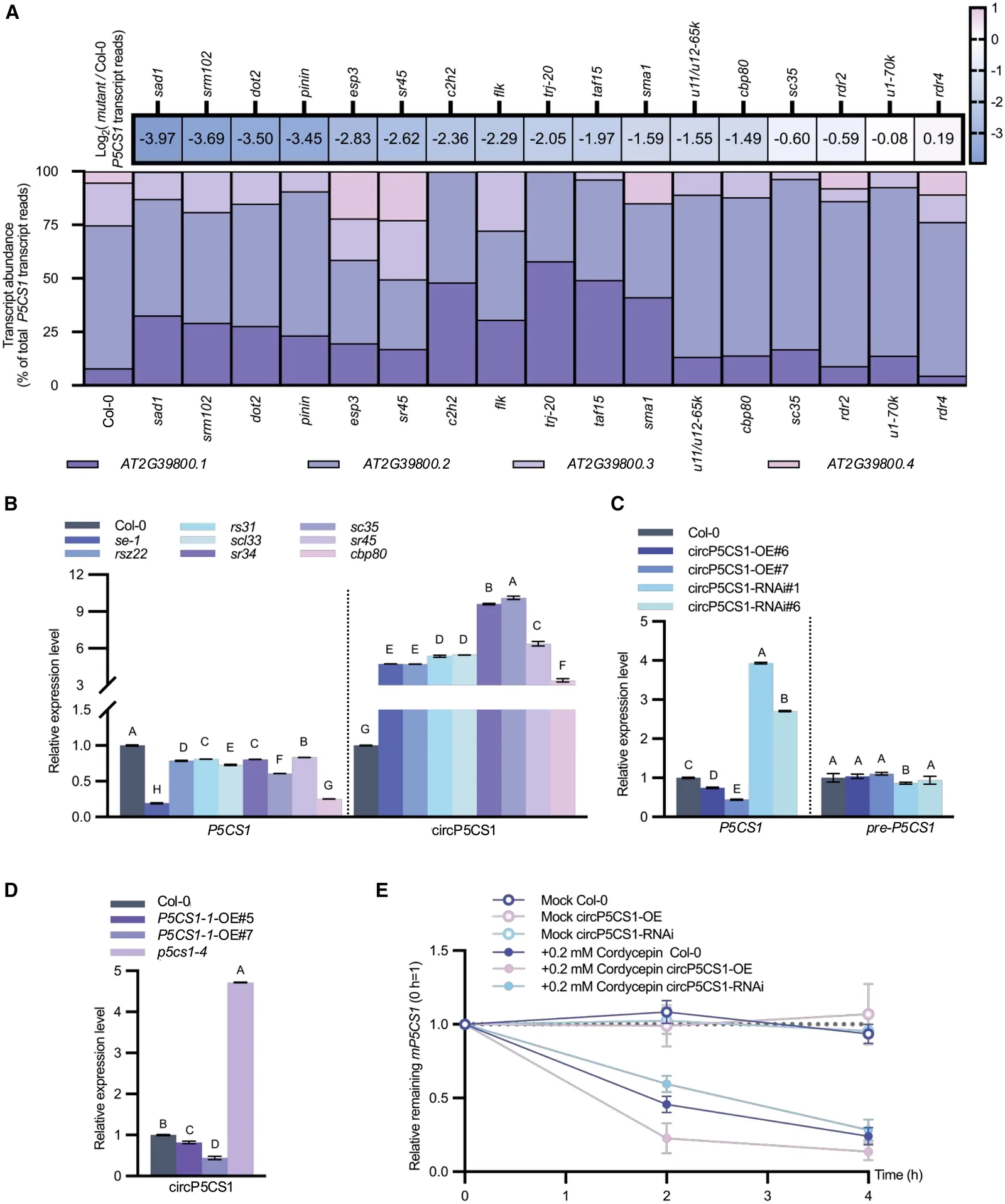
Bidirectional expression patterns of circP5CS1 and *P5CS1* under genetic perturbation. **(A)** RNA sequencing datasets were reanalyzed to examine the abundance of *P5CS1* isoforms in 17 *Arabidopsis* mutants linked to RNA processing or splicing. The heatmap (top) shows the total fold change in *P5CS1* expression relative to that in Col-0, and the stacked bar plots (bottom) show the relative contributions (%) of isoforms 1–4 across the various genotypes. **(B)** RT–qPCR analysis of circP5CS1 and *P5CS1* expression in eight splicing-factor mutants (*se-1*, *rsz22*, *rs31*, *scl33*, *sr34*, *sc35*, *sr45*, and *cbp80*). The data are presented as mean ± SEM (*n* = 3 biological replicates), with the letters A–H denoting significance (*p* < 0.01, one-way ANOVA followed by Duncan’s test for multiple comparisons). **(C)** Expression levels of *P5CS1* and pre*-P5CS1* in circP5CS1 transgenic lines. Data are presented as mean ± SEM (*n* = 3 biological replicates), and letters A–E denote significant differences (*p* < 0.01, one-way ANOVA followed by Duncan’s test for multiple comparisons). **(D)** Expression of circP5CS1 in *P5CS1* transgenic backgrounds. The data are presented as mean ± SEM (*n* = 3 biological replicates), with the letters A–D representing significance (*p* < 0.01, one-way ANOVA followed by Duncan’s test for multiple comparisons). **(E)** Analysis of P5CS1 transcript stability using a cordycepin assay. Three-week-old leaves from Col-0, circP5CS1-OE, and circP5CS1-RNAi plants were treated with 0.2 mM cordycepin or mock solution. Samples were collected at 0, 2, and 4 h after treatment, and P5CS1 transcript levels were quantified by RT–qPCR. Transcript levels were normalized to the corresponding 0-h value for each genotype and treatment. Data are presented as mean ± SEM (*n* = 3 biological replicates).

Focusing on the link between splicing disruption and circP5CS1 production, we examined eight well-characterized mutants with defects in pre-mRNA splicing, including *se-1* (*SERRATE*), *cbp80* (Cap-binding protein 80), and six SR (serine/arginine)- or SR-like factors (*rs31*, *rsz22*, *sr34*, *sr45*, *sc35*, and *scl33*). All eight mutants exhibited significantly elevated circP5CS1 levels, accompanied by concurrent reductions in P5CS1 mRNA levels, representing the combined expression of all four annotated isoforms (Figure 2B). The presence of transfer DNA (T-DNA) insertions (Supplemental Figure 1A) and effective transcript depletion of these splicing factors were confirmed (Supplemental Figure 1B). This inverse expression pattern supports a model in which circP5CS1 accumulation is enhanced under splicing-defective conditions and is correlated with the transcriptional repression of its host gene, *P5CS1*. This altered balance between circular and linear isoforms may reflect disrupted transcription–splicing coupling, in which impaired spliceosome assembly facilitates the biogenesis of circRNA at the expense of canonical mRNA splicing. These findings support the notion that SR proteins fine-tune pre-mRNA fate through co-transcriptional splicing decisions.

To test whether circP5CS1 modulates P5CS1 transcript levels, we quantified the expression of both P5CS1 mRNA and pre*-P5CS1* in circP5CS1-OE and RNAi lines. Significant downregulation of P5CS1 mRNA was observed in circP5CS1-OE plants, whereas RNAi-mediated knockdown of circP5CS1 led to marked upregulation of P5CS1 transcript levels relative to Col-0 (Figure 2C). By contrast, pre*-P5CS1* expression showed no obvious change in circP5CS1-OE plants compared with Col-0 and was unchanged or slightly reduced in circP5CS1-RNAi plants (Figure 2C). These findings suggest that circP5CS1 suppresses *P5CS1* predominantly through post-transcriptional regulation, potentially involving alternative splice site usage. To further assess whether circP5CS1 affects the stability of P5CS1 transcripts, we performed a cordycepin-based transcriptional inhibition assay. Cordycepin is a transcriptional inhibitor that blocks RNA elongation, enabling the evaluation of mRNA decay rates independently of ongoing transcription. Three-week-old leaves from Col-0, circP5CS1-OE, and circP5CS1-RNAi plants were treated with 0.2 mM cordycepin or mock solution, and samples were collected at 0, 2, and 4 h for RT–qPCR analysis. Under mock conditions, P5CS1 transcript levels remained relatively stable in all genotypes. By contrast, after cordycepin treatment, P5CS1 transcript levels decreased over time in all lines, indicating ongoing transcript degradation (Figure 2E). Notably, the decline was more pronounced in circP5CS1-OE plants than in Col-0, whereas circP5CS1-RNAi plants exhibited a slower decrease (Figure 2E). These results indicate that circP5CS1 promotes the turnover of P5CS1 transcripts, providing direct evidence that circP5CS1 regulates *P5CS1* expression at the level of mRNA stability.

We further examined the potential for reciprocal regulation by analyzing circP5CS1 expression in *P5CS1* OE (the most predominant transcript isoform 1, *P5CS1-1*) (P5CS1-1-OE) and knockout (*p5cs1-4*) lines. Whereas circP5CS1 levels were slightly reduced in P5CS1-1-OE plants, a notable increase was observed in the knockout background (Figure 2D). This decoupling of host gene and circRNA expression implies that circP5CS1 biogenesis is not strictly determined by host gene transcription and may be influenced by additional post-transcriptional mechanisms.

Collectively, these findings suggest a complex post-transcriptional feedback loop between circP5CS1 and its host gene, *P5CS1*, modulated by splicing fidelity and transcript dosage. Although circP5CS1 OE was correlated with reduced P5CS1 mRNA levels, the potential for reciprocal influence should not be excluded. This bidirectional interplay underscores the nuanced regulatory relationship between circRNAs and their parental genes for maintenance of transcriptomic homeostasis when RNA processing is perturbed.

### circP5CS1 functions as a decoy to modulate *ARF6/8* expression in *Arabidopsis*

We next assessed the potential for direct interaction between circP5CS1 and miRNAs through sequence alignment. circP5CS1 contains a single predicted miR167 binding site that matches the four known isoforms of miR167 (a–d) in different patterns (Figure 3A). For miR167a and miR167b, a mismatch was observed at position 9, with wobble pairing at positions 8 and 18. For miR167c, mismatches occurred at positions 1, 8, and 20, with wobble pairings at positions 7 and 17. For miR167d, a mismatch was present at position 9, accompanied by wobble pairings at positions 8, 18, and 21. (Figure 3A). These differences suggest that circP5CS1 preferentially binds miR167a/b.

**Figure 3:**
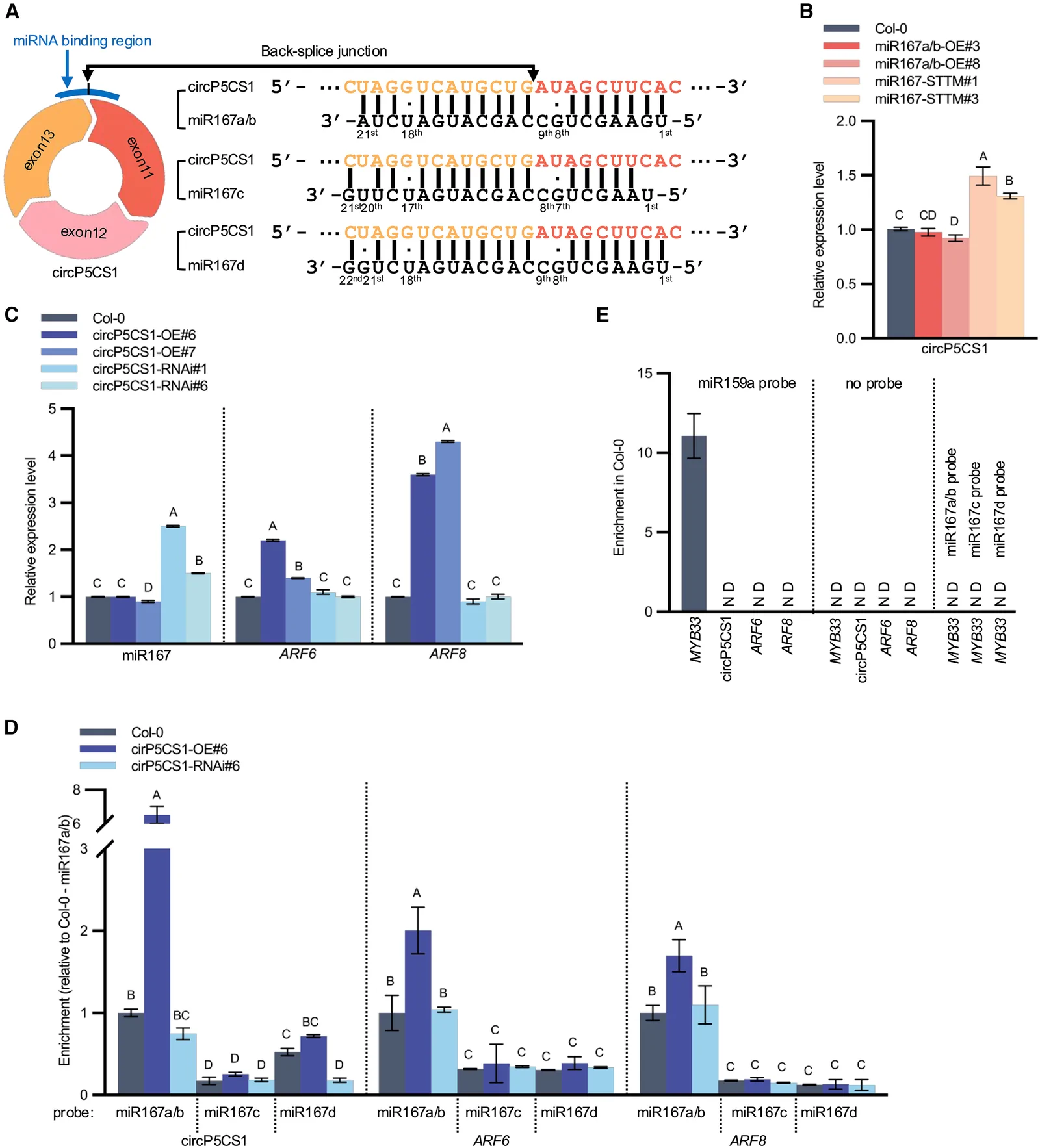
circP5CS1 interacts with miR167 and modulates *ARF6/8* expression in *Arabidopsis*. **(A)** The predicted base-pairing interactions between circP5CS1 and the four isoforms of miR167 (a, b, c, and d) reveal distinct binding patterns. Mismatches between bases are not annotated; a vertical line between bases indicates complementary base pairing, and a dot represents weak pairing. The yellow-highlighted bases in circP5CS1 represent sequences derived from exon 13 of *P5CS1*, and the red-highlighted bases correspond to sequences from exon 11 of *P5CS1*. **(B)** Expression levels of circP5CS1 in Col-0, miR167a/b-OE lines, and miR167-STTM lines. The data are presented as fold change compared with the Col-0 control, with ±SEM based on three biological replicates, and the letters A–D indicate significance (*p* < 0.01, one-way ANOVA followed by Duncan’s test for multiple comparisons). **(C)** Expression of miR167, *ARF6*, and *ARF8* in Col-0, circP5CS1-OE, and circP5CS1-RNAi lines. The data are expressed as fold change relative to Col-0, with error bars representing SEM from three biological replicates, and the letters A–D indicate significance (*p* < 0.01, one-way ANOVA followed by Duncan’s test for multiple comparisons). **(D)** A pull-down assay using biotin-labeled probes for miR167a/b, miR167c, and miR167d was performed in Col-0, circP5CS1-OE, and circP5CS1-RNAi lines to capture circP5CS1, *ARF6*, and *ARF8*, followed by quantification via RT–qPCR. The data are presented as fold enrichment relative to the input, with error bars indicating the SEM from three biological replicates, and the letters A–D indicate significance (*p* < 0.01, one-way ANOVA followed by Duncan’s test for multiple comparisons). **(E)** Negative controls for the pull-down assay shown in **(D)**, performed in the Col-0 background. A biotin-labeled probe for miR159a was used to enrich its known target *MYB33*, and circP5CS1, *ARF6*, and *ARF8*, which are not targets of miR159a, were included to assess potential non-specific binding. To further exclude off-target enrichment, *MYB33* recovery was tested using probes for miR167a/b, miR167c, and miR167d. Pull-downs without probe served as background controls.

Analysis of circP5CS1 expression in Col-0, miR167a/b-OE, and lines expressing an miR167 short tandem target mimic (STTM) that mutes miR167 revealed an inverse relationship between miR167 levels and circP5CS1 abundance. Whereas circP5CS1 levels were unchanged or slightly reduced in miR167a/b-OE lines, they were significantly elevated in the miR167-STTM background (Figure 3B). The altered expression of miR167 in these transgenic lines was confirmed by RT–qPCR (Supplemental Figure 1D). This expression pattern does not indicate that miR167 transcriptionally regulates circP5CS1. Rather, the greater accumulation of circP5CS1 in STTM lines is likely attributable to reduced miRNA–RISC (RNA-induced silencing complex) engagement, which alleviates potential miRNA-induced destabilization and thus enhances the apparent stability of circP5CS1 transcripts. Although circP5CS1 shows strong complementarity to miR167 isoforms, mismatches and wobble pairings at critical positions, particularly near the canonical cleavage site (e.g., position 9), likely prevent AGO1-mediated splicing. Combined with its circular structure, which is inherently resistant to endonucleolytic cleavage, these results suggest that circP5CS1 is more likely to act as a decoy of *ARF6/8* rather than a cleavage target.

Because *ARF6* and *ARF8* are the primary, well-characterized targets of miR167 and are known to play key roles in auxin signaling and immune regulation (Nagpal et al., 2005; Wu et al., 2006; Caruana et al., 2020), we next examined whether circP5CS1 influences the miR167–ARF6/8 regulatory module by competitive binding. ARF6 and ARF8 transcript levels were markedly increased in circP5CS1-OE lines, whereas miR167 levels were unchanged or slightly reduced (Figure 3C). By contrast, circP5CS1-RNAi plants exhibited increased miR167 levels with unchanged expression of *ARF6* and *ARF8* relative to Col-0 (Figure 3C). These patterns support the hypothesis that circP5CS1 can act as a molecular sponge to alleviate miR167-mediated repression of *ARF6/8*.

We further examined the interaction between circP5CS1 and miR167 using biotin-labeled RNA pull-down assays with isoform-specific probes. In the circP5CS1-OE line, circP5CS1 was robustly enriched by the miR167a/b probe, whereas this enrichment was markedly reduced in circP5CS1-RNAi plants (Figure 3D). By contrast, probes targeting miR167c or miR167d consistently showed minimal recovery of circP5CS1 across all genotypes, consistent with their lower predicted binding affinity. A similar pattern was observed for *ARF6* and *ARF8*, which were more strongly enriched in miR167a/b pull-downs from circP5CS1-OE plants, whereas only modest enrichment was detected under circP5CS1-RNAi or control conditions (Figure 3D). To exclude nonspecific interactions, we performed parallel pull-down assays using miR159a probes and a no-probe control. As expected, miR159a probes selectively enriched their *MYB33* target while failing to recover circP5CS1, *ARF6*, or *ARF8* (Figure 3E). No enrichment of any transcript was observed in reactions lacking probes (Figure 3E), further confirming the specificity of the miR167–circP5CS1 interaction. To confirm the specificity of our findings, we performed RNA pull-down assays using miR159a probes in circP5CS1-OE, circP5CS1-RNAi, and circP5CS1-mut-OE (expressing a circP5CS1 variant with mutations in the predicted miR167-binding sites to disrupt the circP5CS1–miR167 interaction) *Arabidopsis* lines. Although the known miR159a target *MYB33* was detected, no binding of circP5CS1, *ARF6*, or *ARF8* was observed in any of these lines (Supplemental Figure 4E).

**Figure 4:**
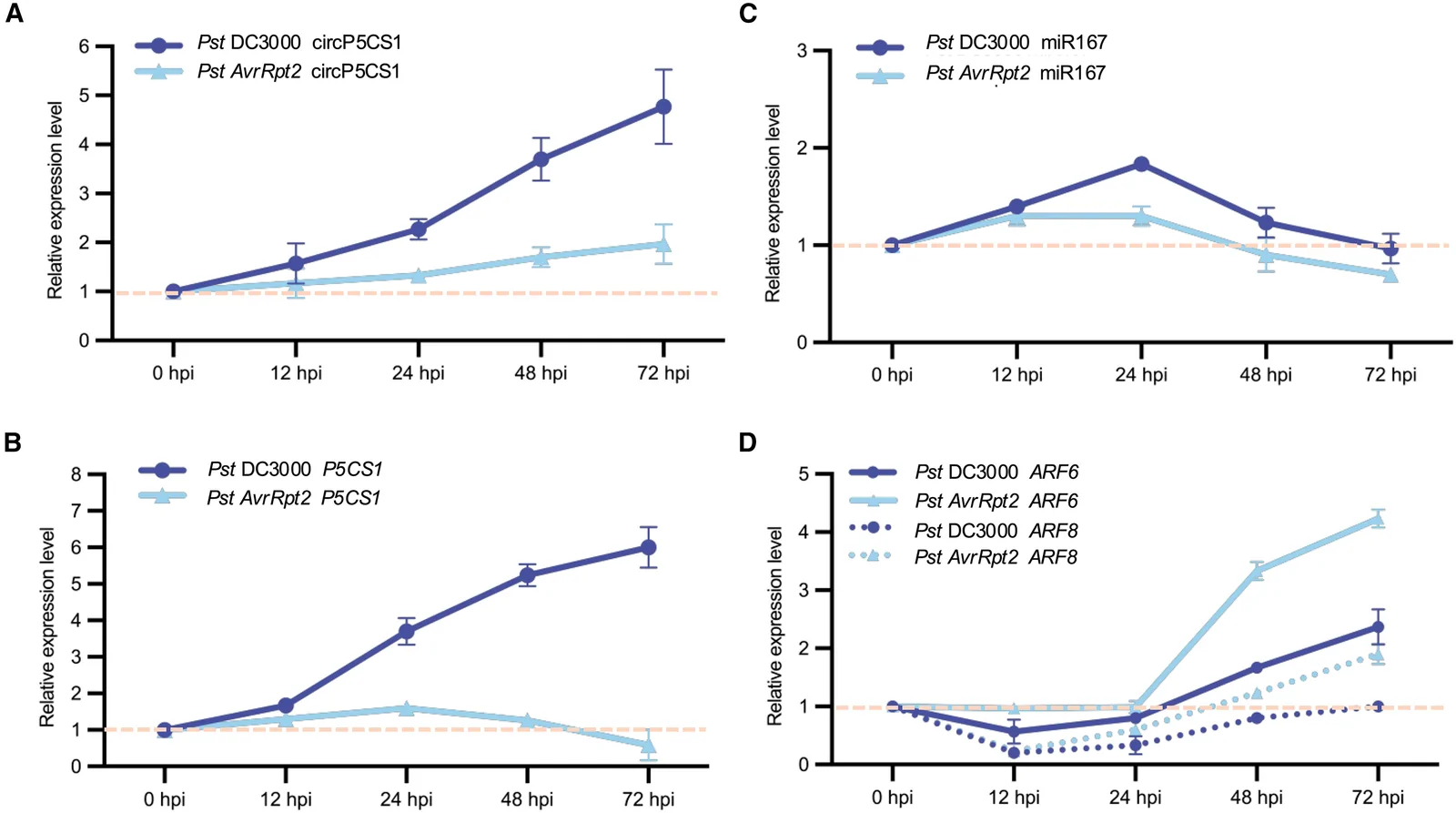
Temporal expression profiles of circP5CS1, *P5CS1*, miR167, and *ARF6/8* in *Arabidopsis* during pathogen infection. **(A–D)** Expression levels of circP5CS1 **(A)**, *P5CS1***(B)**, miR167 **(C)**, and *ARF6/8***(D)** were measured in Col-0 at 0, 12, 24, 48, and 72 hpi with *P. syringae pv. tomato* DC3000 and *P. syringae pv. tomato AvrRpt2*. Data are presented as fold change relative to 0 h. Expression values were normalized to *ACTIN2*. Error bars represent the SEM from three independent biological replicates.

Together, these results demonstrate that circP5CS1 preferentially binds miR167a/b and functions as a decoy to modulate the miRNA-mediated silencing of *ARF6* and *ARF8*. This competitive endogenous RNA (ceRNA)-like mechanism positions circP5CS1 as a critical regulator of auxin signaling components and highlights its potential role in integrating RNA-level regulation with hormonal, developmental, and immune responses in *Arabidopsis*.

To evaluate the evolutionary conservation of this regulatory module, we extended our analysis to rice and rapeseed. Phylogenetic analysis and sequence alignment showed that *P5CS1* and its homologs are conserved across *Arabidopsis*, rice, and rapeseed and that *P5CS1*-like genes in rice and rapeseed also generate predicted circRNAs with putative miR167-binding sites (Supplemental Figure 3). These findings suggest the potential existence of analogous circP5CS1–miR167 modules in other plant species.

### Functional dynamics of the circP5CS1–miR167–ARF6/8–P5CS1 regulatory network under pathogen infection

To investigate the temporal regulation of the circP5CS1–miR167–ARF6/8–P5CS1 network during bacterial infection, we inoculated *Arabidopsis* Col-0 plants with *P. syringae pv. tomato* DC3000 or *P. syringae pv. tomato AvrRpt2*. Transcript levels of elements from the circP5CS1 regulatory module were quantified by RT–qPCR at 0, 12, 24, 48, and 72 h post-infection (hpi). circP5CS1 expression progressively increased under both treatments, with a notably higher and more sustained induction in response to *P. syringae pv. tomato* DC3000, particularly at later infection stages (Figure 4A). *P5CS1* expression showed a similar pattern, with sustained upregulation throughout *P. syringae pv. tomato* DC3000 infection but only a transient increase followed by a decline at 72 hpi upon infection with *P. syringae pv. tomato AvrRpt2* (Figure 4B). Interestingly, circP5CS1 and *P5CS1* were both upregulated during pathogen infection (Figure 4A and 4B), a pattern distinct from their inverse relationship under genetic or splicing perturbation (Figure 2). This suggests that pathogen-triggered transcriptional activation may transiently override circP5CS1-mediated repression, indicating a context-dependent regulatory mode (see discussion). miR167 levels peaked at 24 hpi under both treatments, with stronger and more prolonged induction observed in plants infected with *P. syringae pv. tomato* DC3000 (Figure 4C). This temporal pattern mirrored early circP5CS1 accumulation, suggesting a potential feedback relationship. As miR167 levels declined after 48 hpi, ARF6 and ARF8 transcript levels gradually recovered, especially under *P. syringae pv. tomato AvrRpt2* infection. Consistent with their roles as miR167 targets, *ARF6* and *ARF8* showed an expression profile opposite to that of miR167, characterized by early repression and a late-stage rebound (Figure 4D). Notably, *ARF6* exhibited more pronounced recovery than *ARF8*, and both were expressed at higher levels in response to *P. syringae pv. tomato AvrRpt2* than to *P. syringae pv. tomato* DC3000, suggesting differential relief from miRNA-mediated repression in distinct immune contexts. Collectively, these findings delineate a dynamic and multilayered immune-regulatory network in which circP5CS1 acts both as a miR167 decoy to modulate *ARF6/8* expression and as a conditional suppressor of its host gene, *P5CS1*. This dual regulatory role highlights the capacity of circP5CS1 to coordinate auxin signaling and proline metabolism, which are mediated by ARF6/8 and P5CS1, respectively, potentially contributing to the fine-tuning of immune responses in *Arabidopsis*.

### The circP5CS1–miR167–P5CS1 module regulates proline-associated immune responses under pathogen challenge

Building on observed pathogen-responsive expression patterns, we next sought to determine whether the circP5CS1–miR167–P5CS1 regulatory module directly influences immune function. To this end, we challenged transgenic *A. thaliana* lines with altered expression of circP5CS1, miR167a/b, and *P5CS1-1* with *P. syringae pv. tomato* DC3000 or *P. syringae pv. tomato AvrRpt2* to evaluate responses associated with basal and effector-triggered immunity. circP5CS1-OE and miR167-STTM lines exhibited more severe chlorotic lesions (Figure 5A) and higher bacterial loads (Figure 5B), whereas circP5CS1-RNAi, miR167a/b-OE, and P5CS1-1-OE plants had reduced disease symptoms and enhanced resistance relative to wild-type controls. Thus, downregulation of circP5CS1 or upregulation of miR167 or *P5CS1* enhanced defense against both pathogen strains.

**Figure 5:**
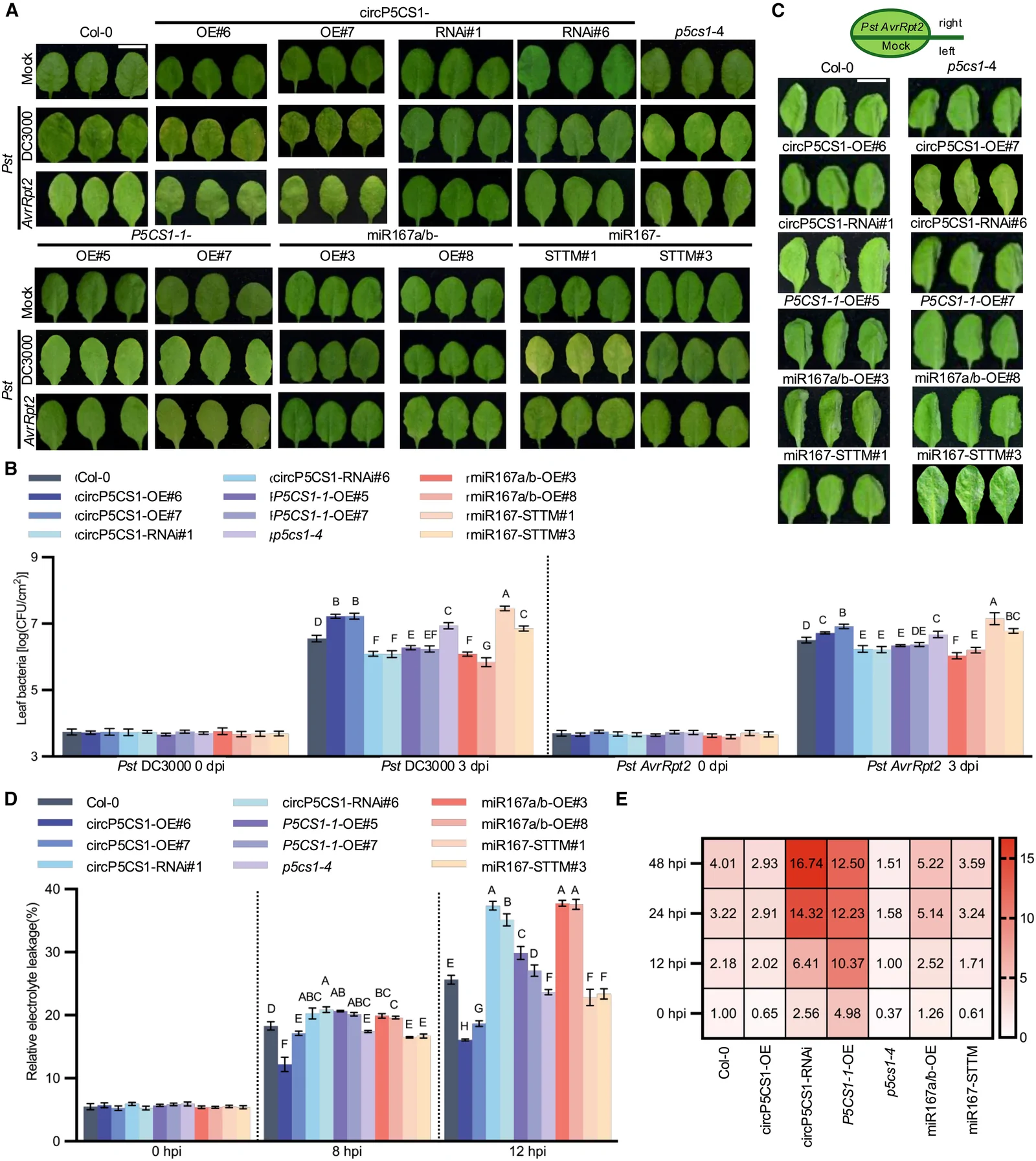
Functional outcomes of the circP5CS1–miR167–P5CS1 regulatory module in *Arabidopsis* immune and metabolic responses. **(A–E)** Functional assays were performed in Col-0 and transgenic lines (OE or RNAi/T-DNA/STTM of circP5CS1, *P5CS1*, or miR167). Data are presented as mean ± SEM from at least three independent biological replicates, and the letters A–D indicate significance (*p* < 0.01, one-way ANOVA followed by Duncan’s test for multiple comparisons). **(A)** Disease symptoms were assessed at 0 and 3 dpi with *P. syringae pv. tomato* DC3000 and *P. syringae pv. tomato AvrRpt2.* Scale bars: 1 cm. **(B)** Bacterial growth was quantified as CFU/cm^2^ at 0 and 3 dpi with *P. syringae pv. tomato* DC3000 and *P. syringae pv. tomato AvrRpt2* in plant leaves. **(C)** The half-leaf HR was evaluated at 12 hpi with *P. syringae pv. tomato AvrRpt2*. Scale bars: 1 cm. **(D)** Electrolyte leakage was measured at 0, 8, and 12 hpi with *P. syringae pv. tomato AvrRpt2*, as a proxy for membrane damage. **(E)** Free proline content was determined at 0, 12, 24, and 48 hpi with *P. syringae pv. tomato DC3000* to assess stress-induced metabolic changes across genotypes.

To further evaluate immune activation, particularly during ETI, we assessed visible HR symptoms and membrane integrity. Strong HR responses were observed in circP5CS1-RNAi, miR167a/b-OE, and P5CS1-1-OE lines, whereas circP5CS1-OE and *p5cs1-4* plants exhibited attenuated HR phenotypes (Figure 5C). Electrolyte leakage assays corroborated these observations (Figure 5D), revealing significantly higher ion leakage in HR-active lines, indicative of enhanced membrane disruption and more intense immune responses.

Given the role of *P5CS1* in proline biosynthesis, we next examined whether the immune phenotypes were correlated with changes in proline metabolism. In Col-0 plants, proline levels increased gradually during infection. Notably, *P5CS1-1*-OE and circP5CS1-RNAi lines showed significantly elevated proline accumulation, whereas circP5CS1-OE and *p5cs1-4* plants exhibited lower levels (Figure 5E). miR167a/b-OE promoted proline accumulation, whereas miR167-STTM suppressed it. These results support a model in which circP5CS1 inhibits *P5CS1* by sequestering miR167, thereby modulating stress-related metabolic outputs. Meanwhile, the circP5CS1–miR167–P5CS1 module also integrates proline metabolism with immune responses, contributing to coordinated defense regulation during both basal and effector-triggered immunity in *Arabidopsis*.

To further characterize the downstream physiological and signaling consequences associated with altered proline accumulation, we next examined ROS production and hormone-related responses. Proline-dependent ROS assays revealed that ROS production was lower in circP5CS1-OE and *p5cs1-4* plants than in Col-0, whereas the responses of circP5CS1-mut-OE were comparable to those of Col-0 (Supplemental Figure 5A). Notably, exogenous proline treatment enhanced ROS production across all genotypes, supporting a role for proline availability in the modulation of ROS-associated immune responses.

Given the established interplay between proline metabolism and hormone signaling, we next quantified endogenous levels of indole-3-acetic acid (IAA), SA, and jasmonic acid (JA) after *P. syringae pv. tomato* DC3000 infection. circP5CS1-OE plants exhibited higher IAA but lower SA accumulation than Col-0, whereas circP5CS1-RNAi plants showed the opposite trends (Supplemental Figure 5B). By contrast, JA levels showed only minor variation across genotypes.

Consistent with these hormone profiles, analysis of hormone-related gene expression revealed that auxin-associated genes (*ARF6*, *ARF8*, IAA-inducible 19 [*IAA19*], and flavin-containing monooxygenase-like protein YUCCA6 [*YUC6*]) were generally upregulated in circP5CS1-OE plants, whereas SA-related genes (isochorismate synthase 1 [*ICS1*], pathogenesis-related gene 1 [*PR1*], and gh3-like defense gene 1 [*GDG1*]) were upregulated in circP5CS1-RNAi plants (Supplemental Figure 5C). JA-related genes (lipoxygenase 2 [*LOX2*], allene oxide synthase [*AOS*], and oxophytodienoate reductase 3 [*OPR3*]) exhibited relatively modest changes.

Together, these results provide further mechanistic support for the theory that the circP5CS1–miR167–P5CS1 module coordinates proline metabolism with ROS production and hormone signaling, thereby contributing to the regulation of immune responses under pathogen challenge.

## Discussion

### circP5CS1 acts as an *ARF6/8* decoy that modulates auxin signaling

circRNAs are increasingly recognized as pivotal regulators in post-transcriptional gene networks, particularly because of their ability to function as miRNA sponges (Memczak et al., 2013; Chen, 2016; Panda, 2018). In this study, we identified circP5CS1, a circRNA derived from the *P5CS1* locus via backsplicing of exons 11–13, as a central node in the miR167–ARF6/8 regulatory module. Interestingly, circP5CS1 abundance also varied upon miR167 OE or knockdown (Figure 3B), which may reflect feedback regulation within the circP5CS1–miR167–ARF6/8 module rather than direct transcriptional control. Because circP5CS1 functions primarily as a sponge for miR167, changes in miR167 levels likely influence circP5CS1 indirectly by affecting *P5CS1* transcription, splicing dynamics, or RNA processing factors. circP5CS1 OE led to reduced miR167 abundance and a corresponding upregulation of *ARF6/8*, whereas RNAi-mediated knockdown of circP5CS1 resulted in miR167 accumulation and *ARF6/8* repression (Figures 3C and 6). These findings support a classic ceRNA model in which circP5CS1 acts as a miRNA sponge to attenuate miR167-mediated silencing (Memczak et al., 2013; Panda, 2018; Chen, 2020). Interestingly, ARF6 and ARF8 transcript levels were not reduced in circP5CS1-RNAi plants despite elevated miR167 levels (Figure 3C), suggesting that basal miR167-mediated repression of *ARF6/8* in Col-0 plants is already near saturation. Consequently, additional miR167 accumulation may not further reduce transcript levels, possibly owing to compensatory transcriptional regulation or predominant translational inhibition mechanisms. Consistent with this notion, the comparable recovery of ARF6/8 transcripts between circP5CS1-RNAi and Col-0 plants in miR167 pull-down assays (Figure 3D) suggests that a modest increase in miR167 fails to significantly enhance its binding to *ARF6/8*.

**Figure 6:**
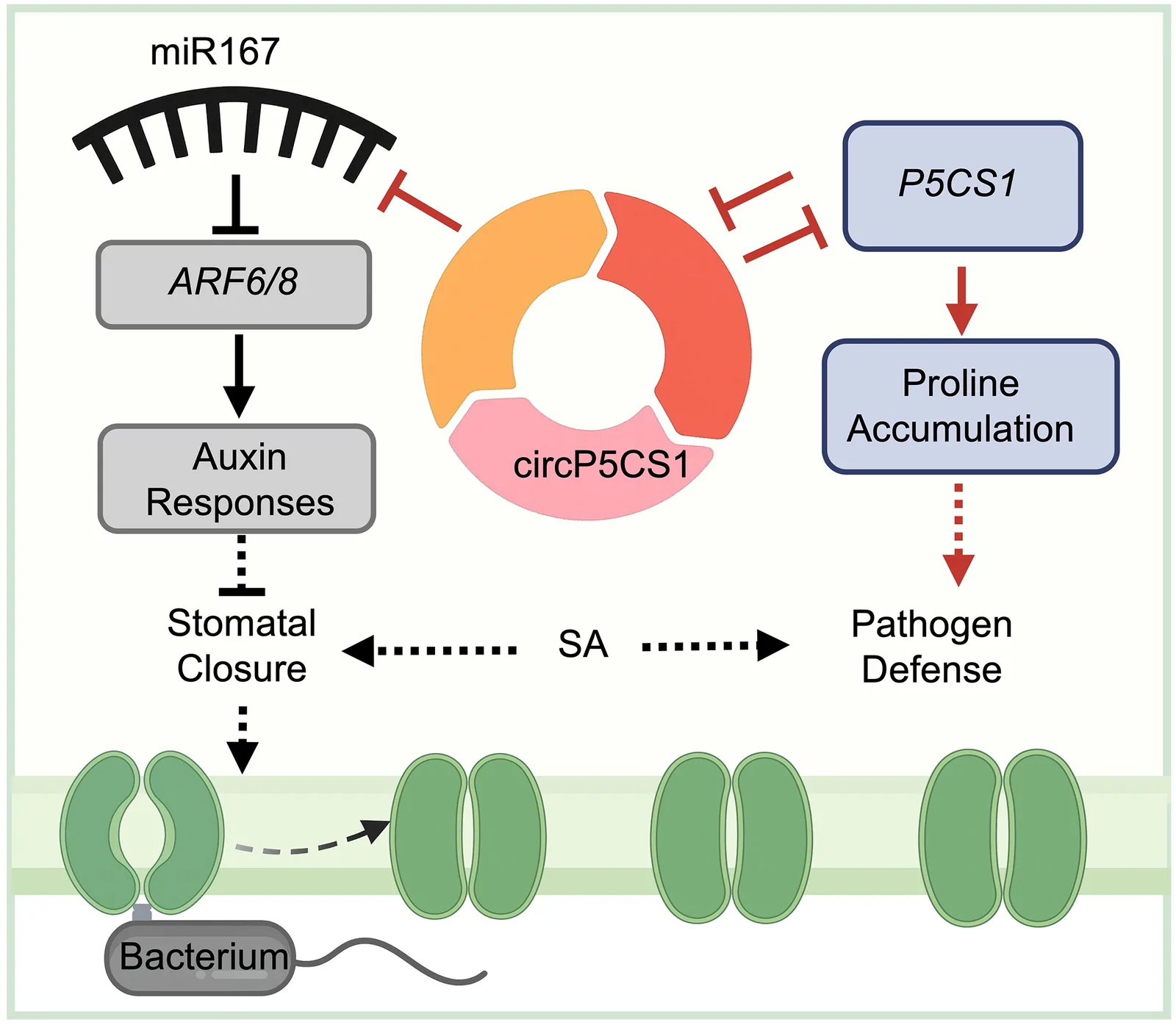
Working model of the circP5CS1–miR167 regulatory network in *Arabidopsis*. circP5CS1 functions as a molecular decoy for miR167, thereby alleviating miR167-mediated repression of *ARF6* and *ARF8* and modulating auxin signaling-associated immune responses. In parallel, circP5CS1 negatively regulates the expression of its host gene *P5CS1*, leading to altered proline accumulation during pathogen infection. Together, these pathways contribute to the fine-tuning of immune responses in *Arabidopsis*. Arrows indicate positive regulatory relationships, and T-shaped lines indicate inhibitory effects. Red lines represent the major regulatory relationships experimentally supported in this study, and black lines indicate indirect, proposed, or previously reported regulatory associations. Solid lines indicate experimentally supported relationships identified in this study, whereas dashed lines represent proposed or indirect regulatory associations. This mechanism complements and extends the miR167–SA/auxin model proposed by Caruana et al. (2020).

RNA pull-down assays revealed a highly conserved binding site at the backsplice junction of circP5CS1 that showed strong complementarity to miR167a/b but not miR167c/d, owing to greater mismatches and wobble base-pairing (Figure 3A and 3D). This binding selectivity indicates that its sponge activity is selectively tuned toward isoforms with more stable pairing. In addition to development (Nagpal et al., 2005; Okushima et al., 2005; Wu et al., 2006), miR167-mediated repression of *ARF6/8* has also been shown to enhance SA-dependent defense, promoting stomatal closure and resistance to *P. syringae* (Caruana et al., 2020). Conversely, derepression of *ARF6/8* activates JA biosynthesis by LOX2 and AOS, favoring a JA-dominant response that supports growth-compatible immunity (Nagpal et al., 2005; Kazan and Manners, 2009). Our findings suggest that, by sponging miR167a/b, circP5CS1 may bias the hormonal equilibrium toward JA-mediated pathways, potentially modulating the balance between growth (Supplemental Figure 2) and defense under pathogen pressure. However, our current study does not provide direct evidence of JA accumulation or altered expression of JA biosynthesis genes such as *LOX2* or *AOS*. Therefore, the proposed involvement of JA signaling is inferred from the well-established regulatory role of ARF6/8 in activating JA-related pathways (Nagpal et al., 2005; Caruana et al., 2020) and warrants further validation in future studies.

Similar mechanisms have been described in other crops, suggesting that circRNA–miRNA–mRNA networks may be broadly involved in stress responses. In rice, circANK has been shown to bind miR398b, thereby suppressing resistance to *Xanthomonas oryzae* pv. *oryzae* (Liu et al., 2025), and in maize, circZmMED16 regulates flowering time by interacting with metabolic enzymes such as ZmAPS1 (Tang et al., 2025). This regulatory circuit may represent a conserved mechanism across plant species, although functional validation is needed. Notably, our identification of circRNAs with putative miR167-binding sites in rice and rapeseed is based on computational prediction and should be considered preliminary. These observations suggest the possible existence of analogous circRNA–miRNA interactions in other plant species rather than demonstrating functional conservation. Integrative transcriptomic studies in rice, particularly those investigating the *M. oryzae* pathosystem, have identified over 2900 high-confidence circRNAs, with 636 specifically induced by pathogen infection (Fan et al., 2020). These stress-responsive circRNAs form ceRNA networks by binding miRNAs and modulating downstream mRNA targets involved in immunity. These converging findings underscore a broader regulatory paradigm in which circRNAs serve as noncoding hubs, buffering miRNA activity to fine-tune gene expression programs that reconcile developmental plasticity with immune prioritization. In our study, we confirmed the functional importance of the circP5CS1–miR167 interaction by generating a circP5CS1 mutant with disrupted miR167-binding sites. The mutant exhibited responses comparable to those of wild-type plants, in contrast to circP5CS1-OE lines, indicating that miR167-binding capacity is required for circP5CS1-mediated regulation. In addition, expression of the ARF6/8 downstream targets *LOX2* and *AOS* further supports the role of this module in coordinating immune-related gene expression. Future work will focus on defining the downstream regulatory network of ARF6/8, including potential direct targets and chromatin-level regulation.

### circP5CS1 and *P5CS1* exhibit reciprocal regulation at transcriptional and post-transcriptional levels

The formation of circP5CS1 does not result in novel linear P5CS1 transcripts lacking any exons (Figure 1B), indicating that circP5CS1 is generated via canonical backsplicing but not through exon-skipping-dependent alternative splicing. However, levels of specific P5CS1 transcript isoforms (*AT2G39800.1–AT2G39800.4*) varied significantly among wild-type and RNA-processing or RNA-splicing mutants (Figure 2A). These isoforms encode transcripts with slightly different coding products. *AT2G39800.3* and *AT2G39800.4* are almost identical except for 9 nt in exon 13. *AT2G39800.1* differs at the 5′ and 3′ UTRs, and *AT2G39800.2* features a distinct first exon corresponding to the 3′ region of exon 4 in transcript 1 and a different 5′ UTR with otherwise identical sequences. Isoform dynamics may therefore represent one mechanism through which circP5CS1 modulates *P5CS1* expression at the transcriptional level.

SR family members are known to affect splice-site selection and spliceosome assembly, which are critical for circRNA biogenesis (Tanabe et al., 2007; Long and Caceres, 2009; Reddy et al., 2013). In all tested mutants (*rs31*, *rsz22, sr34*, *sr45*, *sc35*, and *scl33*), increased expression of circP5CS1 was associated with a significant reduction in P5CS1 transcript levels (Figure 2A and 2B), indicating a robust inverse relationship between circular and linear isoforms of *P5CS1*. This pattern is consistent with a precursor competition model in which increased backsplicing limits the pool of pre-mRNA available for canonical exon–exon splicing, thereby reducing host mRNA output (Ashwal-Fluss et al., 2014). This inverse relationship suggests a co-transcriptional splicing competition model whereby backsplicing of circP5CS1 precludes the formation of linear P5CS1 mRNA, as reported in animal systems (Zhang et al., 2014). Our findings extend this conserved mechanism to plants, where it remains to be fully explored. Splicing occurs co-transcriptionally in many systems (Jabre et al., 2019; Zhu et al., 2020), and our results support a role for circRNA biogenesis in the modulation of host-gene expression at the transcription level.

Interestingly, RNAi-mediated silencing of circP5CS1 significantly restored P5CS1 transcript levels. This finding suggests that circP5CS1 also regulates the expression of its host gene at the post-transcriptional level, potentially by influencing mRNA stability, splicing efficiency, or the availability of shared RNA-processing factors. Consistent with this possibility, our results also indicate that circP5CS1 promotes the decay of P5CS1 transcripts, supporting a role in the modulation of mRNA stability. This provides direct evidence that circP5CS1 represses *P5CS1* expression at the post-transcriptional level by accelerating transcript turnover (Figure 2E). Similar regulatory relationships between circular and linear RNAs have been reported in both animals (Ashwal-Fluss et al., 2014) and plants (Lu et al., 2015). Consistent with this model, P5CS1-OE and knockout lines revealed that *P5CS1* itself also influences circP5CS1 accumulation (Figure 2D), supporting a bidirectional feedback mechanism between the circRNA and its host gene. Further corroborating this hypothesis, Philips et al. (2020) demonstrated that *Arabidopsis* mutants defective in RNA-binding or RNA-processing factors such as CBP80, SERRATE, or SR34 all showed greater accumulation of circRNAs, including many derived from stress-related genes, accompanied by perturbed host-mRNA levels. Collectively, these findings support a model in which circP5CS1 also negatively regulates *P5CS1* at the post-transcriptional level, likely through competition for splicing factors, alterations in splice site choice, or buffering of transcript levels under physiological stress. Although circP5CS1 typically acts to dampen *P5CS1* expression under steady-state or splicing-defective conditions (Figure 2), its simultaneous induction during pathogen challenge (Figure 4) likely reflects a context-dependent regulatory balance. Pathogen-induced transcriptional activation of *P5CS1* may override or precede circP5CS1-mediated repression, leading to the concurrent accumulation of both linear and circular transcripts. Stress conditions may also promote circRNA biogenesis, further contributing to higher levels of circP5CS1. In this context, circP5CS1 may function as a fine-tuning mechanism to prevent excessive accumulation of P5CS1 transcripts. This interplay highlights a dynamic balance between transcriptional activation and circRNA-mediated post-transcriptional regulation, exemplifying a noncoding RNA–host gene feedback loop that integrates metabolic and immune demands.

### circP5CS1 integrates hormone signaling and metabolic adaptation to orchestrate plant immunity

*P5CS1* is the rate-limiting enzyme in the glutamate pathway of proline biosynthesis (Szabados and Savouré, 2010), and rapid proline accumulation has been shown to contribute to adaptive defense (Kishor et al., 2005; Verslues and Sharma, 2010). As a compatible solute, proline contributes to osmotic adjustment, membrane stabilization, and macromolecular protection under drought, salinity, and oxidative stress (Szabados and Savouré, 2010). More importantly, proline metabolism influences cellular redox homeostasis through nicotinamide adenine dinucleotide phosphate (NADP^+^/NADPH) cycling, modulates ROS levels, and interacts with multiple hormonal signaling networks, including those of SA, JA, and abscisic acid (Cecchini et al., 2011; Liang et al., 2013; Signorelli, 2016). In this context, increased proline biosynthesis via *P5CS1* has been shown to enhance resistance to *P. syringae* and *Botrytis cinerea* (Qamar et al., 2015). Our study showed that circP5CS1-OE plants, miR167-STTM lines, and the *p5cs1-4* mutant contained significantly less proline and showed increased disease susceptibility, whereas circP5CS1-RNAi, miR167-OE, and P5CS1-1-OE plants showed elevated proline levels and enhanced resistance (Figure 5). These findings support a functional link connecting the circP5CS1–miR167–P5CS1 module, proline accumulation, and immune adaptation.

Proline-mediated immune regulation is intimately associated with ROS homeostasis and hormone-signaling crosstalk. Previous studies have demonstrated that proline catabolism via the PRODH–P5C cycle promotes respiratory burst oxidase homolog-dependent ROS bursts, thereby amplifying mitogen-activated protein kinase cascades and activating SA-responsive defense pathways (Cecchini et al., 2011; Qamar et al., 2015). Consistent with this framework, alterations in circP5CS1 levels significantly affected ROS production in our proline-dependent ROS assays (Supplemental Figure 5), particularly under exogenous proline treatment, reinforcing the role of proline metabolism in ROS-associated defense responses. In addition, hormone profiling revealed that circP5CS1 modulates the balance between auxin and SA signaling: circP5CS1-OE plants exhibited higher IAA but lower SA accumulation, whereas circP5CS1-RNAi plants showed the opposite trend. These changes were further supported by the expression patterns of hormone-related genes. Together, these findings provide direct evidence that circP5CS1 links proline metabolism with downstream ROS production and hormone signaling, thus contributing to the coordination of growth and immune responses during pathogen challenge.

We also observed distinct developmental phenotypes associated with altered expression of circP5CS1, *P5CS1*, and miR167 (Supplemental Figure 2). Under short-day conditions (10-h light/14-h dark), rosettes of circP5CS1-OE and P5CS1-1-OE plants were slightly smaller than those of Col-0. By contrast, miR167a/b-OE plants exhibited previously reported phenotypes—including reduced bolting height—at 6 weeks but not at 4 weeks (Wu et al., 2006), and circP5CS1-RNAi, miR167-STTM, and the *p5cs1-4* plants showed rosette phenotypes similar to those of Col-0. These morphological changes likely reflect shifts in auxin and JA signaling as well as altered proline accumulation, further linking the circP5CS1–miR167–ARF6/8 axis to growth–defense trade-offs.

Our data also show that circP5CS1-OE plants exhibit derepression of *ARF6*/*8*, suggesting broader shifts in hormonal regulation. Although our study did not directly test activation of the auxin or JA pathways, prior work has shown that proline and auxin pathways can intersect at multiple levels (Liang et al., 2013; Qamar et al., 2015) and that ARF6/8 mediate auxin- and JA-associated transcriptional responses involved in developmental and immune trade-offs (Kazan and Manners, 2009; Caruana et al., 2020). These converging lines of evidence raise the possibility that circP5CS1 coordinates metabolic and hormonal reprogramming in response to biotic stress. Our findings position circP5CS1 as a central regulatory node linking noncoding RNA regulation, proline homeostasis, and immune competence. Interestingly, *P5CS1* undergoes alternative splicing, generating transcript isoforms that may have different functional implications. Previous studies have demonstrated that *P5CS1* isoforms vary in their activity, with some splice variants producing less active or nonfunctional proteins that could influence proline biosynthesis and stress adaptation (Kesari et al., 2012). In our study, we observed that the alternative splicing of *P5CS1* was influenced by RNA-processing mutations. Specifically, isoforms 1 and 2 predominated in RNA processing mutants, and isoforms 3 and 4 were largely absent or present at low levels across most backgrounds. These findings suggest that splicing-related factors may regulate *P5CS1* expression in an isoform-dependent manner, a possibility that could have functional consequences for proline accumulation and immune responses. Given the critical role of proline in stress adaptation and immune responses, we speculate that differences in the accumulation of *P5CS1* isoforms in response to environmental stress may influence plant immunity. These observations highlight the complex regulatory mechanisms controlling proline metabolism and the importance of isoform-specific regulation in coordinating immune responses. By modulating *P5CS1* expression and proline accumulation, circP5CS1 influences both metabolic buffering and defense prioritization (Figure 6). Although both ARF6/8 and P5CS1 participate in circP5CS1-mediated immunity, our data do not support a direct, hierarchical regulatory relationship between them. ARF6/8 primarily function as auxin response factors, whereas P5CS1 regulates proline biosynthesis; their interplay is likely mediated through convergent hormonal and metabolic signaling pathways rather than a linear upstream–downstream relationship. Given the conservation of miR167–ARF modules and proline metabolism across plant species, the mechanistic insights revealed here may be broadly applicable, offering opportunities for enhancing crop stress resilience through targeted manipulation of the circRNA–miRNA–host gene axis. The observed variations in rosette architecture and inflorescence structure among circP5CS1, miR167, and *P5CS1* transgenic lines further underscore the integrative role of this regulatory module in coordinating developmental and immune processes (Figure 5 and Supplemental Figure 2).

In summary, this study identifies circP5CS1 as a central regulatory node that integrates post-transcriptional and metabolic control to fine-tune immune responses in *A. thaliana* (Figure 6). By acting as a miR167 sponge and a repressor of its host gene, circP5CS1 coordinates auxin signaling via ARF6/8 and proline biosynthesis, thereby linking hormonal crosstalk with metabolic plasticity under pathogen challenge. The bifunctional regulatory module under *P. syringae pv. tomato* DC3000 and *P. syringae pv. tomato AvrRpt2* infection highlights the capacity of circP5CS1 for context-dependent immune modulation. These findings extend the functional scope of plant circRNAs beyond miRNA sequestration, positioning circP5CS1 as a bifunctional hub that orchestrates immune prioritization through integrated signaling pathways. Nevertheless, key mechanistic questions remain, such as whether circP5CS1 physically interacts with RNA-binding proteins or RISC components and how it suppresses *P5CS1* transcriptionally or via spliceosomal competition. Furthermore, the potential compartmentalization of circP5CS1 in stress granules or D-bodies and its role in subcellular regulatory specificity require further investigation. Future work that combines RNA structure profiling, protein–RNA interaction mapping, and spatiotemporal imaging will be essential to characterize these layers of regulation. Ultimately, the multifaceted role of circP5CS1 offers a conceptual framework for exploring circRNA-mediated immune adaptation and provides new molecular entry points for the engineering of stress-resilient crops.

## Methods

### Plant mutant materials

This study used a collection of *A. thaliana* mutant lines, including *p5cs1-4*, *cbp80*, *se-1*, and several splicing-related mutants (*rsz22*, *rs31*, *sr34*, *scl33*, *sc35*, and *sr45*), all in the Col-0 background. For most experiments, plants were grown in controlled growth chambers at 22°C–24°C under 16-h light/8-h dark conditions, although plant phenotypes were observed after growth under 10-h light/14-h dark conditions.

The following lines were obtained from the Arabidopsis Biological Resource Center (The Ohio State University, USA). (1) *p5cs1-4* (*AT2G39800*, SALK_63517C), harboring a T-DNA insertion in exon 14 of *P5CS1*. The primers used for RT–qPCR to detect *P5CS1* expression were in exons 11 and 12 of the *P5CS1* gene. Despite the T-DNA insertion in exon 14, these primers can detect low levels of residual *P5CS1* expression in the *p5cs1-4* mutant. (2) *cbp80* (*AT2G13540*, SALK_024285C), containing a T-DNA insertion in intron 13. (3) *se-1* (*AT2G27100*, CS3257), an X-ray-induced loss-of-function allele with a 7-bp deletion near the C-terminal region of the *SERRATE* coding sequence.

The following splicing-factor mutants were kindly provided by Prof. Luqing Zheng (Nanjing Agricultural University, China): (1) *rsz22* (*AT4G31580*, SALK_012172C) with a T-DNA insertion in the 5′ UTR, (2) *rs31* (*AT3G61860*, SALK_021332C) with an insertion in exon 5, (3) *sr34* (*AT1G02840*, SALK_106067C) with an insertion in exon 9, (4) *scl33* (*AT1G55310*, SALK_058566C) with an insertion in exon 6, (5) *sc35* (*AT5G64200*, SALK_124563C) with an insertion in exon 3, and (6) *sr45* (*AT1G16610*, SALK_063528C) with a T-DNA insertion in the 5′ UTR.

Genomic DNA was extracted using the cetyltrimethylammonium bromide (CTAB) method, and homozygous insertion alleles were verified by PCR with gene-specific and LBb1.3 primers. Total RNA was isolated from rosette leaves using the TRIzol reagent, and transcript levels were analyzed by RT–qPCR. RT–qPCR data were analyzed using one-way ANOVA, followed by Duncan’s test for multiple comparisons. *p* < 0.01 was considered statistically significant. Data are presented as mean ± SEM (*n* = 3 biological replicates). All primers used in this study are listed in Supplemental Table 1.

### Generation of transgenic lines

The construct for circP5CS1-OE was carefully designed to encourage the backsplicing of *P5CS1* exons 11, 12, and 13. To augment the efficiency of circularization, we incorporated two endogenous flanking intronic sequences: a 79-bp fragment upstream of exon 11 (extracted from intron 10) and an 85-bp fragment downstream of exon 13 (sourced from intron 13). To further encourage circRNA formation, we inserted 597-bp reverse complementary sequences derived from the first intron of *AT2G39720*, selected at random, at both termini of the construct. The final construct was incorporated into the pCAMBIA vector, driven by the CaMV 35S promoter. An identical strategy was employed for the circP5CS1-mut-OE construct, except that five nucleotides within the predicted miR167-binding sites were mutated to disrupt the circP5CS1–miR167 interaction (Supplemental Figure 4A).

To specifically attenuate circP5CS1, we devised an amiRNA that targeted the unique backsplice junction (exons 11–13) using the WMD3 amiRNA platform (http://wmd3.weigelworld.org), which was subsequently cloned into the pEarleyGate 202 vector, also driven by the 35S promoter.

In a parallel effort, amiRNAs aimed at miR167a/b were constructed using the same platform to facilitate their OE. To silence miR167, we used the STTM strategy, designed to sequester endogenous miR167 molecules, adhering to the protocol established by Teotia and Tang (2017); the construct was ultimately cloned into the pFGC5941 vector, which features the 2× 35S promoter. *P5CS1* was overexpressed by integrating its full-length coding sequence into the pEarleyGate 202 vector, also under the 35S promoter, together with a C-terminal FLAG epitope tag for detection.

All constructs were introduced into *A. thaliana* Col-0 plants via *Agrobacterium tumefaciens* strain GV3101 using the floral dip transformation method. Transgenic T1 seedlings were selected on Murashige and Skoog medium supplemented with Basta or kanamycin, depending on the resistance cassette used. Homozygous T3 lines with single-locus insertions were confirmed through genomic PCR and RT–qPCR using the primer sequences listed in Supplemental Table 1. RT–qPCR data were analyzed using one-way ANOVA followed by Duncan’s test for multiple comparisons. Significance was determined at a threshold of *p* < 0.01, and the results are presented as mean ± SEM (*n* = 3 biological replicates).

### RNA extraction and RT–qPCR

Total RNA was extracted from rosette leaves of 3-week-old *A. thaliana* plants using the TRIzol reagent (Invitrogen, Waltham, MA, USA) according to the manufacturer’s protocol. Genomic DNA was removed by DNase I treatment (Takara, China) to ensure RNA integrity for downstream applications.

For quantitative analysis of gene expression, 1 μg of DNase-treated RNA was reverse transcribed using HiScript III All-in-One RT SuperMix (Vazyme, R333), which contains both oligo(dT) and random hexamer primers. The resulting cDNA was used as a template for RT–qPCR performed with Taq Pro Universal SYBR qPCR Master Mix (Vazyme, Q712) on an RT–qPCR detection system.

Transcript levels were normalized to those of the reference gene *ACTIN2* (*AT3G18780*), and relative expression values were calculated using the 2^−ΔΔCt^ method. Each biological replicate was analyzed in technical triplicate.

RT–qPCR data were analyzed by one-way ANOVA followed by Duncan’s test for multiple comparisons. The significance threshold was set at *p* < 0.01, and the results are expressed as mean ± SEM (*n* = 3 biological replicates). All primer sequences used in this study are provided in Supplemental Table 1.

### Pathogen inoculation and disease assays

*P. syringae pv. tomato* DC3000 and *P. syringae pv. tomato AvrRpt2* were cultured overnight at 28°C in King’s B medium and then resuspended in 10 mM MgCl_2_ to the desired optical density for plant inoculation.

To assess disease phenotypes and bacterial proliferation, a bacterial suspension of 1 × 10^5^ colony-forming units (CFU)/ml was syringe infiltrated into fully expanded rosette leaves of 3-week-old *A. thaliana* plants. Disease symptoms were visually evaluated and photographed at 0 and 3 days post-inoculation (dpi) in Col-0, circP5CS1-OE and RNAi lines, miR167a/b-OE and STTM lines, and P5CS1-1-OE and *p5cs1-4* mutant lines.

For analysis of proline content, leaves from each genotype were harvested at 0, 12, 24, and 48 hpi with *P. syringae pv. tomato* DC3000 or *P. syringae pv. tomato AvrRpt2* and processed according to the protocol described under proline quantification.

To profile gene-expression dynamics, infected Col-0 leaves were collected at 0, 12, 24, 48, and 72 hpi for RT–qPCR analysis of circP5CS1, P5CS1, miR167, ARF6, and ARF8 transcript levels.

For the HR assay, a suspension of *P. syringae pv. tomato AvrRpt2* (1 × 10^7^ CFU/ml) was infiltrated into half of a rosette leaf at 12 hpi. Ion leakage measurements were performed separately, as detailed in the following section.

For quantification of bacterial growth, leaf discs were collected at 3 dpi, ground in 10 mM MgCl_2_, serially diluted, and plated on King’s B agar containing rifampicin. CFUs were counted after 48 h of incubation at 28°C.

All experiments were independently repeated at least three times using biological replicates.

### Ion leakage assays

Electrolyte leakage was measured to assess cell membrane integrity as an indicator of the HR associated with *P. syringae pv. tomato AvrRpt2*. Leaf discs (0.5 cm in diameter) were excised from fully expanded rosette leaves of *A. thaliana*.

Discs were infiltrated with a suspension of *P. syringae pv. tomato AvrRpt2* (1 × 10^7^ CFU/ml) and collected at 0, 8, and 12 hpi. For each genotype and time point, 6 discs were floated in 5 ml of ultrapure water in sterile 15-ml centrifuge tubes and incubated at room temperature with gentle shaking. After 3 h, the initial conductivity was recorded using a conductivity meter (Bante Instruments, China).

To determine total electrolyte content, samples were boiled at 100°C for 25 min and then cooled to room temperature. Final conductivity was measured, and ion leakage was calculated using the following formula: ion leakage (%) = (initial conductivity/total conductivity) × 100. All measurements were performed using three independent biological replicates of each genotype and time point.

### Proline quantification

Proline content was quantified using a colorimetric assay kit (Proline Assay Kit, E-BC-K177-M, Elabscience, Wuhan, China) according to the manufacturer’s instructions. For each genotype, 100 mg of fresh rosette leaves were collected at 0, 12, 24, and 48 hpi.

Leaf samples were briefly rinsed with ultrapure water, blotted dry, and homogenized in 900 μl of extraction reagent I, maintaining a 1:9 (w/v) tissue-to-buffer ratio. The homogenates were centrifuged at 10,000× g for 15 min at 4°C, and the supernatants were collected for analysis.

Aliquots of the clarified extracts were incubated with the kit’s chromogenic reagent at 95°C for 30 min. After cooling to room temperature, absorbance was measured at 520 nm using a microplate reader (Infinite E Plex, Tecan, Switzerland). Proline concentrations were calculated from a standard curve generated from serial dilutions of the provided L-proline standard and normalized to sample fresh weight (per 0.1 g tissue). All measurements were performed in technical triplicate with at least three independent biological replicates for each genotype and time point.

### miRNA pull-down assay

To assess the direct interaction between circP5CS1 and miR167, a biotin-based miRNA pull-down assay was performed using streptavidin-coated magnetic beads (BeyoMag, Beyotime, China) and 5′-biotinylated miRNA probes specifically targeting miR167a/b, miR167c, and miR167d. A probe for miR159a was included as a negative control. All probes were synthesized by Tsingke Biotech (China). Total RNA was extracted from *A. thaliana* Col-0, circP5CS1-OE, and circP5CS1-RNAi lines using TRIzol reagent (Invitrogen). RNA quality was verified by NanoDrop spectrophotometry, and only samples with an A260/280 ratio close to 2.0 were used.

Probe coupling to magnetic beads

Magnetic beads (75 μl; 10 mg/ml) were washed 3 times with 1× binding and washing buffer I (5 mM Tris–HCl [pH 7.5], 0.5 mM EDTA, 1 M NaCl, 0.05% Tween 20). Beads were then incubated with 100 pmol of 5′-biotinylated probe (prepared in 100 μl, 1 μM) for 30 min at room temperature with gentle rotation, ensuring a molar excess relative to streptavidin binding capacity. Unbound probes were removed by washing three more times with the same buffer. To minimize nonspecific binding and RNase contamination, beads were washed twice with diethyl pyrocarbonate (DEPC)-treated 0.05 M NaCl (2 min per wash) and once with DEPC-treated 0.1 M NaCl. The cleaned, probe-loaded beads were resuspended in 2× binding and washing buffer to the original volume for downstream hybridization.

RNA hybridization and washing

Each hybridization reaction contained 10 μg of total RNA in 500 μl binding buffer incubated with the probe-coupled beads for 2 h at 4°C with gentle rotation. For normalization, 10% of the input RNA (1 μg) was reserved before hybridization and stored at −80°C. After hybridization, beads were magnetically separated and washed 4 times with 500 μl of pre-chilled washing buffer (4°C, 5 min per wash) to remove nonspecifically bound RNA.

RNA elution and quantification

Bound RNA was extracted by adding 1 ml of TRIzol reagent directly to the beads and incubating at room temperature for 5 min, followed by the addition of 200 μl chloroform. After vortexing and a 3-min incubation, samples were centrifuged at 12,000 *g* for 15 min at 4°C. The aqueous phase was transferred to a new tube, mixed with isopropanol, and incubated at −20°C for 30 min to 2 h. RNA was pelleted by centrifugation (12,000 *g*, 10 min, 4°C), washed once with 70% ethanol, air-dried, and resuspended in 15–20 μl RNase-free water.

Reverse transcription was performed using HiScript III All-in-One RT SuperMix (Vazyme, R333), followed by quantitative PCR using Taq Pro Universal SYBR qPCR Master Mix (Vazyme, Q712). Relative enrichment was calculated as the ratio of pull-down signal to input for each target RNA.

### *P5CS1* transcript stability assay

To investigate the stability of P5CS1 transcripts, a cordycepin-based transcriptional inhibition assay was performed. Three-week-old leaves from Col-0, circP5CS1-OE, and circP5CS1-RNAi plants were treated with 0.2 mM cordycepin or mock solution (ddH_2_O, double-distilled water) for 0, 2, and 4 h. Leaf disks (5 mm in diameter) were cut and placed, adaxial side up, in individual wells of a 96-well microtiter plate. Each well contained 150 μL of cordycepin solution or mock solution. After the treatment period, RNA was extracted using the TRIzol reagent according to the manufacturer’s instructions. RNA (1 μg) was reverse transcribed using HiScript III All-in-One RT SuperMix (Vazyme, R333) and diluted 10-fold for subsequent RT–qPCR. RT–qPCR was performed using Taq Pro Universal SYBR qPCR Master Mix (Vazyme, Q712) on an RT–qPCR detection system. The expression of *P5CS1* was normalized to the corresponding 0-h time point for each genotype and treatment. Data are presented as mean ± SEM (*n* = 3 biological replicates).

### ROS production assay

ROS production was measured using a previously described luminol-based assay (Smith and Heese, 2014) with minor modifications. Leaf discs (5 mm in diameter) were collected from 3-week-old plants (Col-0, circP5CS1-OE, *p5cs1-4*, and circP5CS1-mut-OE) and cut into two equal halves using a sharp razor blade to increase the exposed surface area, thereby improving reproducibility. Each half leaf disc was floated, adaxial side up, in a 96-well plate containing 150 μl ddH_2_O and incubated overnight (approximately 20 h) at 22°C under continuous light to minimize wounding responses.

The incubation solution was then replaced with 150 μl of 10 mM proline, and samples were incubated for 4 h under the same conditions. Prior to elicitation, an elicitation solution was prepared containing *P. syringae pv. tomato* DC3000 bacterial cells (optical density 600 = 0.1), luminol, and horseradish peroxidase. Specifically, 20 μl of 500× horseradish peroxidase stock and 20 μl of 500× luminol stock were added to 10 ml of bacterial suspension.

Immediately before measurement, the incubation solution was carefully removed and replaced with 100 μl of elicitation solution. Luminescence was recorded immediately using a 96-well-plate luminometer at 1-min intervals over 80 min. ROS production was expressed as relative luminescence units, and total ROS output was quantified as the area under the curve. Each experiment included 12 biological replicates.

### Hormone measurement and gene expression analysis

Plants (Col-0, circP5CS1-OE, and circP5CS1-RNAi) were inoculated with *P. syringae pv. tomato* DC3000 (optical density 600 = 0.0005), and leaf samples were collected at 0, 6, 12, 24, and 48 hpi. Endogenous levels of IAA, SA, and JA were quantified using commercial assay kits according to the manufacturer’s instructions.

For gene expression analysis, total RNA was extracted and reverse transcribed into cDNA. RT–qPCR was performed to determine the expression levels of auxin-related genes, SA-related genes, and JA-related genes. Transcript levels were normalized to that of *ACTIN2* and calculated using the 2^−ΔΔCt^ method.

All hormone and gene expression data were normalized to Col-0 at 0 h. Heatmaps based on normalized values were generated to visualize changes across genotypes and time points. Each experiment included three biological replicates.

### Tools and data analysis

Gene expression graphs were generated using Prism 10.

All data were analyzed using Student’s *t*-test or one-way ANOVA followed by Duncan’s test for multiple comparisons. *p* < 0.01 was considered statistically significant. All experiments were performed with at least three biological replicates, and each experiment had three technical replicates. Statistical significance was assessed using SPSS Statistics.

Phylogram: UniProt alignment platform (https://www.uniprot.org/align).

Sequence alignment: ESPript (https://espript.ibcp.fr/ESPript/cgi-bin/ESPript.cgi) (Robert and Gouet, 2014).

miRNA sequences: miRbase (https://www.mirbase.org/).

circOs05t0455500-02(12) and circCDY20482(12,11,10): PlantCircRNA (https://plant.deepbiology.cn/PlantCircRNA/) (He et al., 2025).

## Funding

This research was supported by aYangtze River Delta Scientific and Technological Innovation Community Collaborative Research (Basic Research) Project (24CSJ140200) to H.Z. and the 10.13039/501100004761Natural Science Foundation of Hainan Province (grant 325MS018) to L.W.

## Acknowledgments

We thank Dr. Jason W. Reed and Dr. Lanjie Zheng for valuable suggestions and for providing the *mARF6* and *mARF8* mutant seeds. We are also grateful to Dr. Luqing Zheng for generously sharing a panel of *Arabidopsis* splicing-factor mutants. No conflict of interest is declared.

## Author contributions

L.W. and H.Z. conceived and designed the research. L.W., X. Sun, R.W., X. Shi, L.X., Z.Y., C.T., L.L., and B.G. performed most of the experiments. L.W., H.L., X. Sun, and H.Z. analyzed the data, and L.W. and H.Z. wrote the manuscript. All authors read and approved the final version of the manuscript.

## Supplemental information

Document S1. Supplemental Figures S1–S5 and Supplemental Table S1

Document S2. Article plus supplemental information
