# Supplementary material for: The circular RNA circP5CS1 coordinates plant immunity by sequestering miR167 to modulate ARF6/8 signaling and repressing P5CS1-mediated proline biosynthesis in Arabidopsis
Source: Plant Commun. 2026 May 20;7(10):101922. doi: 10.1016/j.xplc.2026.101922 (PMC13681498; doi:10.1016/j.xplc.2026.101922)
Supplement: Supplementary file 1 [file mmc1.pdf]

Plant Communications, Volume *7*

Supplemental information

The circular RNA circP5CS1 coordinates plant immunity by sequestering miR167 to modulate ARF6/8 signaling and repressing P5CS1-mediated proline biosynthesis in *Arabidopsis*

Lin Wang, Xiaoning Song, Ran Wei, Xuechao Shi, Le Xu, Ze Yu, Cheng Tang, Leyao Li, Baohuan Guo, Hongxia Liu, Xiaoyong Sun, and Hongwei Zhao

Supplemental Information

The circular RNA circP5CS1 coordinates plant immunity by sequestering miR167 to modulate *ARF6/8* signaling and repressing *P5CS1*-mediated proline biosynthesis in *Arabidopsis*

Lin Wang*[,1,2,4], Xiaoning Song[2,3], Ran Wei[2,3], Xuechao Shi[2,3], Le Xu[2,3], Ze Yu[2,3], Cheng Tang[2,3], Leyao Li[2,3], Baohuan Guo[2,3], Hongxia Liu[2,3], Xiaoyong Sun*[,4] and Hongwei Zhao*[,2,3]

Affiliations:

[1] College of Life and Environmental Science, Wenzhou University, Wenzhou 325035, China

[2] State Key Laboratory of Agricultural and Forestry Biosecurity, College of Plant Protection, Nanjing Agricultural University, Nanjing, 210095, China.

[3] Key Laboratory of Integrated Management of Crop Disease and Pests, Ministry of Education, Nanjing Agricultural University, Nanjing, 210095, China.

[4] Agricultural Big-Data Research Center, College of Information Science and Engineering, Shandong Agricultural University, Taian 271018, China.

***Correspondence:** Hongwei Zhao (hzhao@njau.edu.cn); Xiaoyong Sun (johnsunx1@yahoo.com); Lin Wang (linwangviolin@gmail.com)

Short Summary: circP5CS1 functions as a regulatory hub linking miR167 signaling, proline metabolism, and immunity in *Arabidopsis*. Mechanistically, circP5CS1 sequesters miR167a/b to modulate *ARF6/8* signaling while repressing its host gene *P5CS1*, thereby coordinating hormone-associated defense responses and proline biosynthesis during pathogen infection.

Extended Figures:

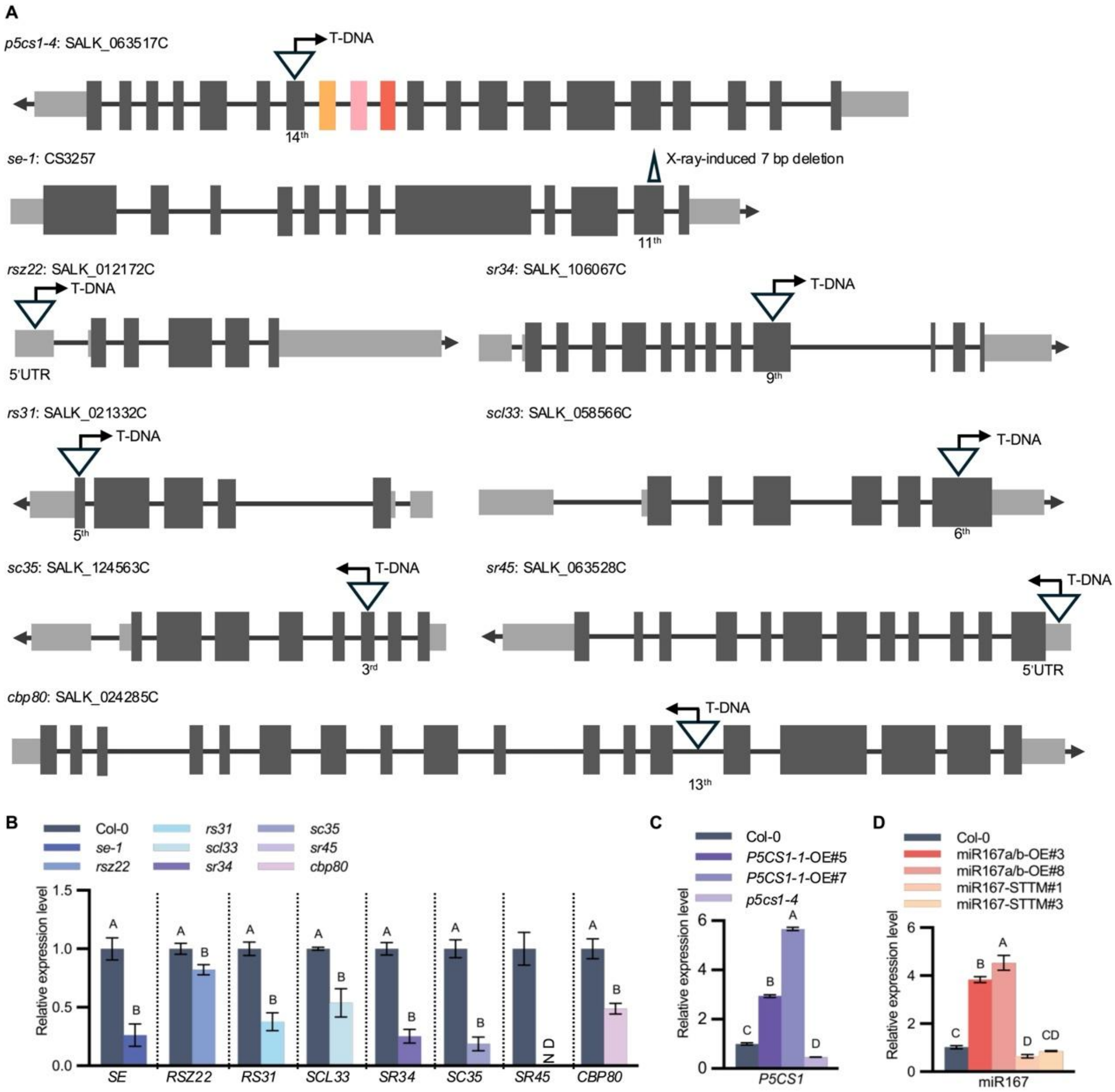

Extended Data Fig.1. Molecular characterization of *P5CS1* and miR167 transgenic lines and splicing factor mutants.

(A) A schematic illustration delineating the T-DNA insertion sites of *p5cs1-4*, *se-1*, *rsz22*, *rs31*, *scl33*, *sr34*, *sc35*, *sr45*, and *cbp80*. The gene models are meticulously derived from TAIR10, while the annotations pertaining to the insertions are informed by T-DNA Express: Arabidopsis Gene Mapping Tool (http://signal.salk.edu/cgi-bin/tdnaexpress).

(B) The expression levels of the corresponding splicing factor genes across each mutant background reveal a significant reduction in transcript levels when juxtaposed with Col-0, thereby affirming the efficacy of gene disruption. The notation ND signifies not detected. The data are presented as means ± SD (n = 3) and the letters A and B indicating significance (P <

0.01, One way ANOVA, Duncan's test).

(C) The expression levels of *P5CS1* in Col-0, *P5CS1-1*-OE, and *p5cs1-4* lines were quantified through qRT-PCR. The data are expressed as means ± SD (n = 3), with letters A-D indicating significance (P < 0.01, One way ANOVA, Duncan's test).

(D) The expression levels of miR167 in Col-0, miR167a/b-OE, and miR167-STTM lines were assessed via qRT-PCR. The data are represented as means ± SD (n = 3), with letters A-D indicating significance (P < 0.01, One way ANOVA, Duncan's test).

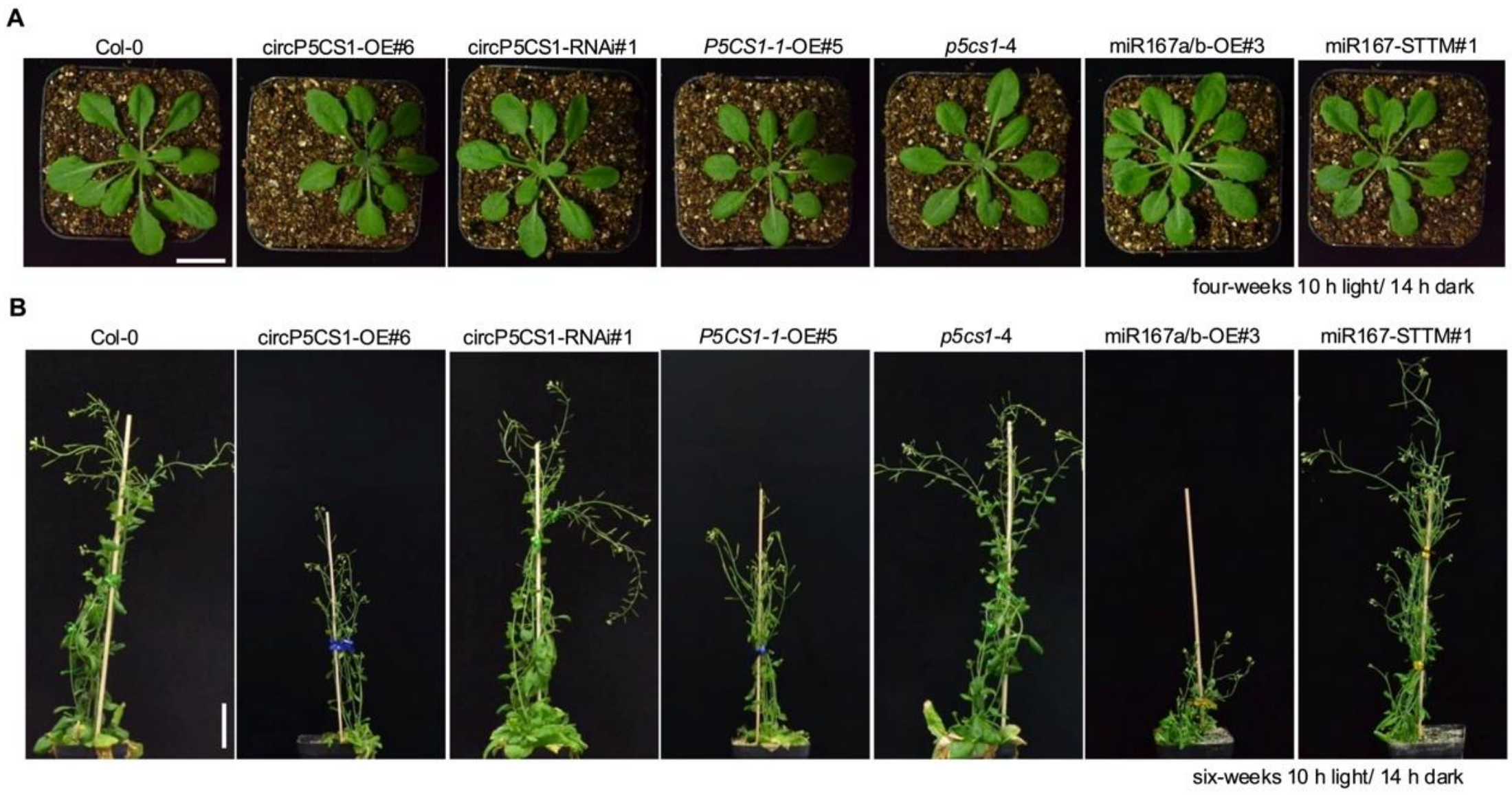

Extended Data Fig.2. Developmental phenotypes of *Arabidopsis* lines with altered circP5CS1, miR167, or *P5CS1* expression.

Arabidopsis thaliana plants grown under short-day conditions (10 h light / 14 h dark). **(A)** 4-week-old. Scale bars = 3 cm. **(B)** 6-week-old. Genotypes from left to right: Col-0, circP5CS1-OE#6, circP5CS1-RNAi#1, *P5CS1-1*-OE#5, *p5cs1-4*. miR167a-OE and miR167-STTM. Scale bars = 5 cm

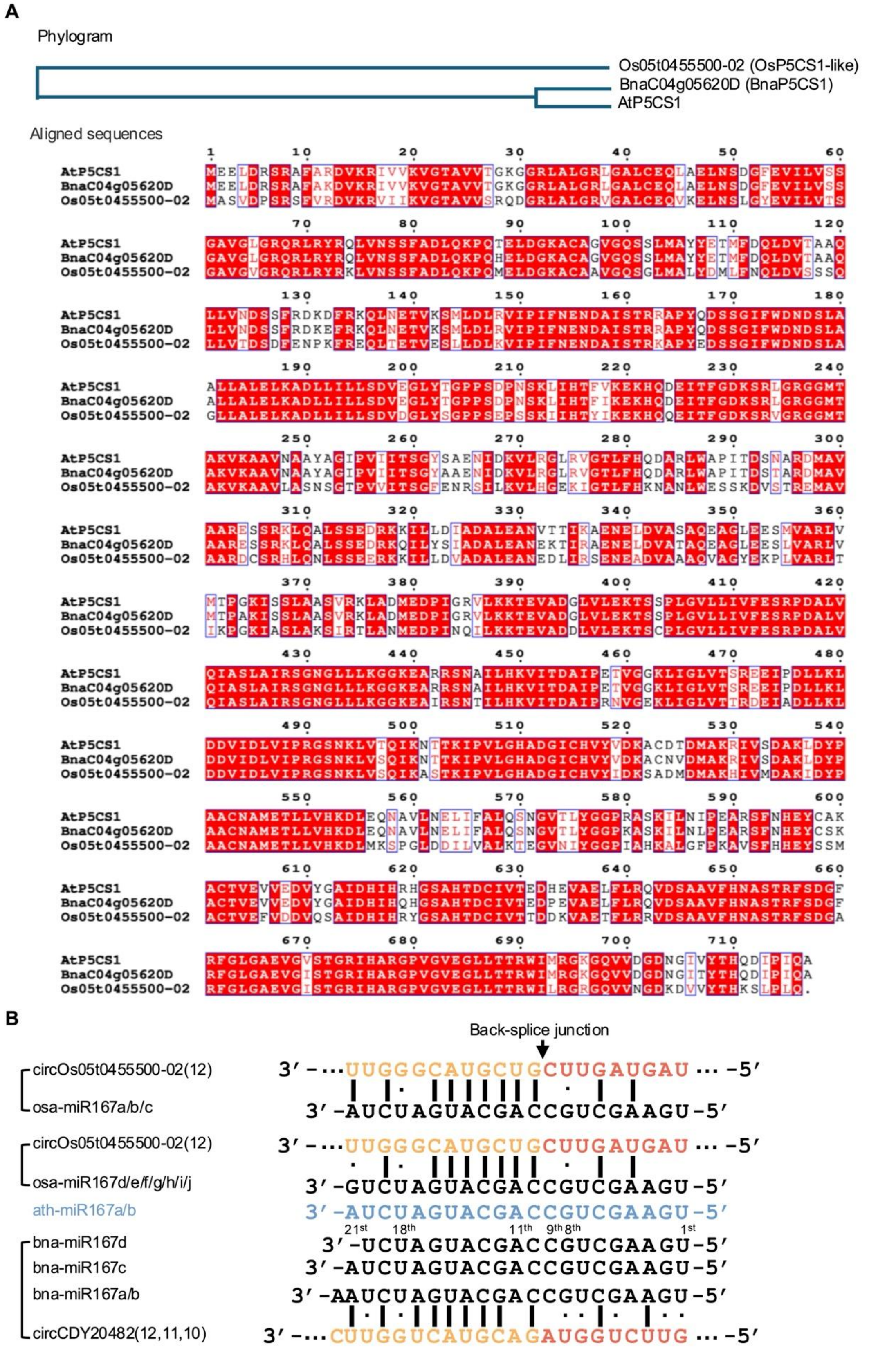

Extended Data Fig.3. Relationships between *P5CS1*-derived circRNAs and miR167 in rice and rapeseed homologous to *Arabidopsis*.

(A) Phylogram of *P5CS1* from *Arabidopsis thaliana*, *P5CS1*-like from Rice（*Oryza sativa*）, and *P5CS1* from Rapeseed（*Brassica napus*）, generated using the UniProt alignment tool (top). The sequence alignment of these three proteins obtained with ESPript (bottom).

(B) Predicted circular RNA candidates: (1) circOs05t0455500-02(12), derived from *O. sativa P5CS1*-like, and its anticipated base-pairing with the ten isoforms of osa-miR167 (a-j). (2) circular RNA circCDY20482(12,11,10), derived from *B. napus P5CS1*, and its anticipated base-pairing with the four isoforms of bna-miR167 (a-d).

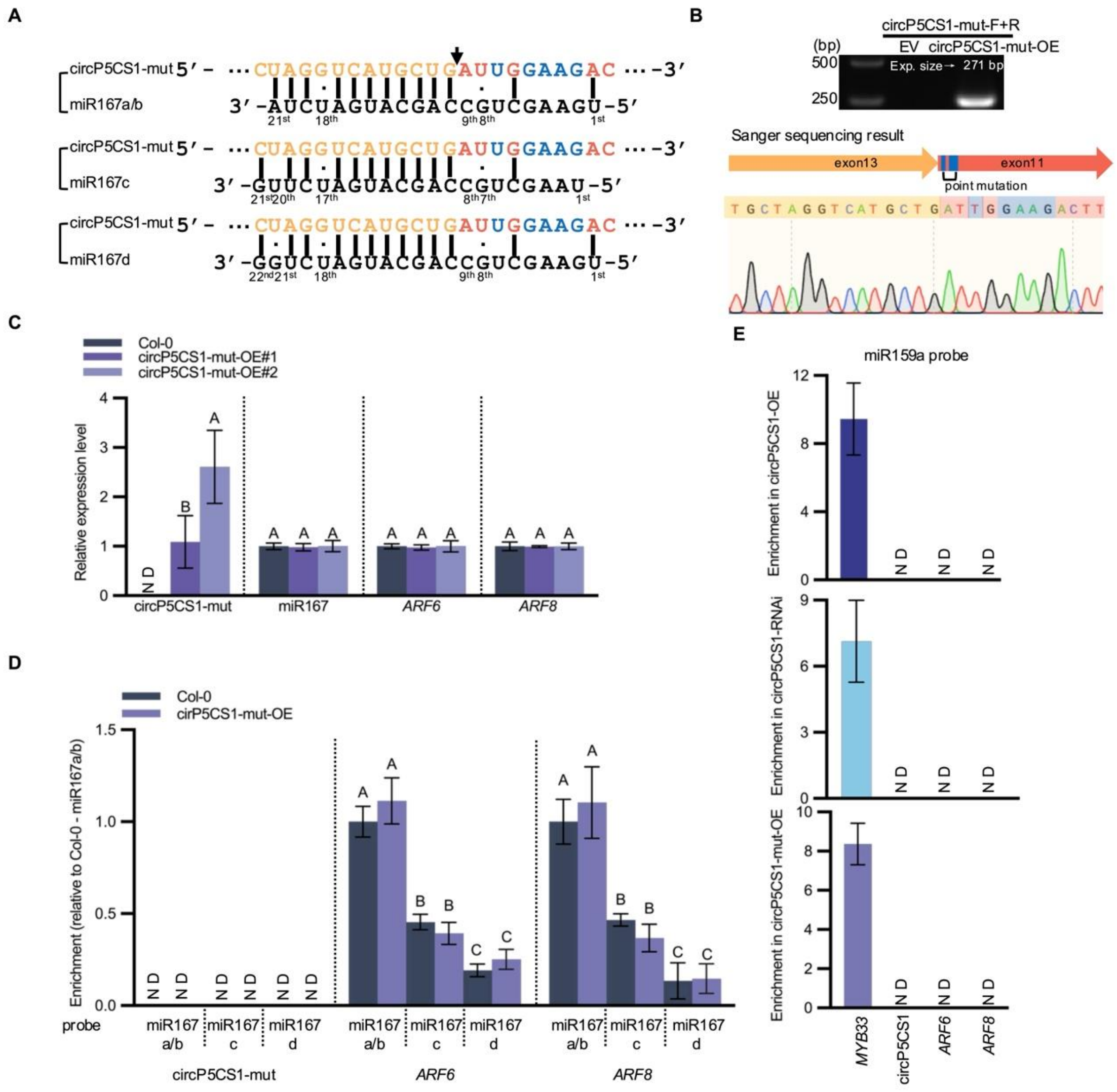

Extended Data Fig.4. Characterization of circP5CS1-mut-OE and its interaction with miR167.

(A) Sequence alignment of circP5CS1-mut with miR167a/b, miR167c, and miR167d, showing mutations at positions 2, 3, 4, 5, and 7 of miR167 (highlighted in blue).

(B) RT-PCR analysis of circP5CS1-mut-OE expression in Nicotiana benthamiana after transient expression, with corresponding Sanger sequencing confirming the expression of mutated circRNA.

(C) RT-PCR analysis of circP5CS1-mut-OE expression in Arabidopsis (Col-0 background), detecting circP5CS1-mut but not the wild-type circP5CS1. Expression of miR167, *ARF6*, and *ARF8* was similar in Col-0 and circP5CS1-mut-OE lines. Data represent the mean ± SEM of 3 biological replicates. Statistical significance was determined using Student's t-test.

(D) RNA pull-down assays in Col-0 and circP5CS1-mut-OE plants using miR167a/b, miR167c, and miR167d probes. No interaction between circP5CS1-mut and miR167 was detected in either line. Data represent the mean ± SEM of 3 biological replicates. Statistical significance was determined using Student's t-test.

(E) RNA pull-down assays in Arabidopsis lines (circP5CS1-OE, circP5CS1-RNAi, circP5CS1-mut-OE) using miR159a probes. *MYB33* was detected as a target of miR159a, but no binding of circP5CS1, *ARF6*, or *ARF8* was observed. Data represent the mean ± SEM of 3 biological replicates. Statistical significance was determined using Student's t-test.

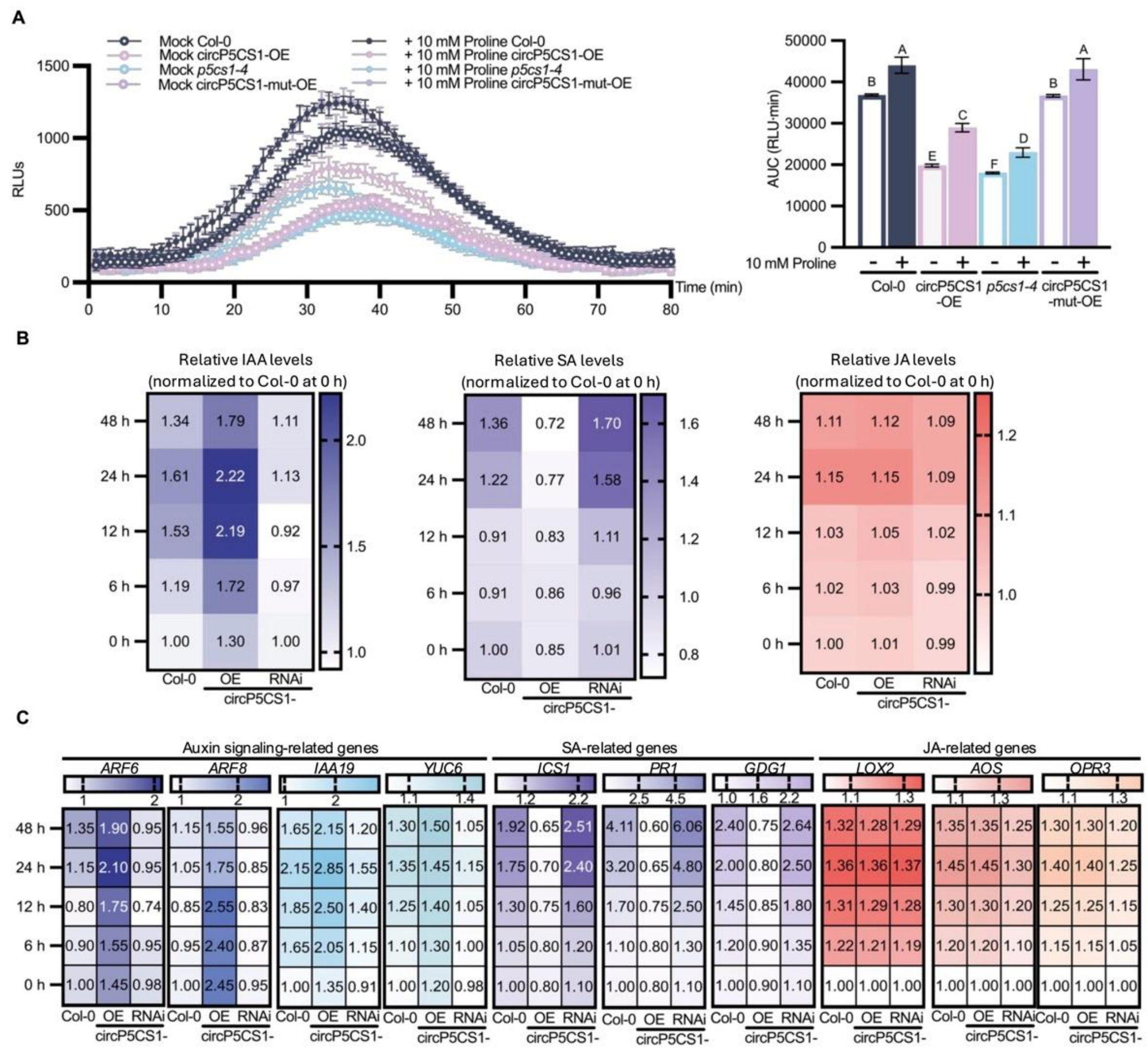

Extended Data Fig.5. circP5CS1 regulates proline-dependent ROS responses and hormone signaling during pathogen challenge.

(A) ROS production kinetics (left) in leaf discs from 3-week-old plants (Col-0, circP5CS1-OE, *p5cs1-4*, and circP5CS1-mut-OE) treated with or without 10 mM proline following *Pst* DC3000 elicitation. Quantification of ROS production (right) as the area under the curve (AUC). n = 12 biological replicates for ROS assays.

(B) Heatmap showing endogenous levels of IAA, SA, and JA in Col-0, circP5CS1-OE, and circP5CS1-RNAi plants at indicated time points after *Pst* DC3000 infection (0, 6, 12, 24 and 48 h). Values were normalized to Col-0 at 0 h. (n = 3 biological replicates)

(C) Heatmap showing expression of hormone-related genes, including auxin-related (*ARF6*, *ARF8*, *IAA19*, *YUC6*), SA-related (*ICS1*, *PR1*, *GDG1*), and JA-related (*LOX2*, *AOS*, *OPR3*) genes in Col-0, circP5CS1-OE, and circP5CS1-RNAi plants at indicated time points after *Pst* DC3000 infection (0, 6, 12, 24 and 48 h). Values were normalized to Col-0 at 0 h. (n = 3 biological replicates).

Extended Data Table 1. Oligonucleotide sequences and references used in this study. This table lists all primers and probes applied for qRT-PCR, miRNA pull-down, and genotyping assays, including sequences targeting circP5CS1, P5CS1, miR167, ARF6/8, and internal controls.

No,	Primer Name	Sequence	Appliaction
1	P5CS1-F1	ATGGAGGAGCTAGATCGTTCAC	RT-PCR/P5CS1-OE construct
2	P5CS1-R2	CTTGTGTAAGATAGCATTTGATCGC	RT-PCR
3	P5CS1-F3	ATAGCTTCACTTGCCATCCG	RT-PCR/ qRT-PCR
4	P5CS1-R4	CAGCATGACCTAGCACAGGG	RT-PCR
5	P5CS1-F5	CTTGATGACGTTATCGATCTTGTG	RT-PCR
6	P5CS1-R6	TTAAGCTTGGATGGGAATGTCCT	RT-PCR
7	P5CS1-F7	GTGGCAGATGGTCTTGTCTTAGAG	RT-PCR
8	P5CS1-R8	CATCGCATTACAGGCTGCTGGATA	RT-PCR/P5CS1-OE construct
9	P5CS1-F9	GTAAGAACAGATTTACAAGCTAGGAGC	qRT-PCR
10	P5CS1-12-intr-F	GTAAGAACAGATTTACAAGCTAGGAGC	qRT-PCR
11	ACTIN 2-F	CAAGCTGTTCTCTCCTTGTAC	RT-PCR/ qRT-PCR
12	ACTIN 2-R	GTAACCTCTCTCTGTAAGGATCTTC	RT-PCR/ qRT-PCR
13	SALK_063517C-LP	CTCTGCAACTTCGTGATCCTC	Identification of T-DNA insertion in the p5cs1-4 mutant
14	SALK_063517C-RP	TGATCACTGATGCAATTCCAG	Identification of T-DNA insertion in the p5cs1-4 mutant
15	SALK_012172C-LP	CCTGCTTTACGGTTAGGGATC	Identification of T-DNA insertion in the rsz22 mutant
16	SALK_012172C-RP	CTCATCCTCAAGTTCACGCTC	Identification of T-DNA insertion in the rsz22 mutant
17	SALK_021332C-LP	ATTATCCCACCTGATGGGAAG	Identification of T-DNA insertion in the rs31 mutant
18	SALK_021332C-RP	AAAGCCCCTTCAAATCTGAAG	Identification of T-DNA insertion in the rs31 mutant
19	SALK_058566C-LP	TCTTCCTCGCCATTTTGTATG	Identification of T-DNA insertion in the scl33 mutant
20	SALK_058566C-RP	AAAAGCTTCCTCCATGAGCTC	Identification of T-DNA insertion in the scl33 mutant
21	SALK_106067C-LP	CATCTGCTTCTTGGCAAGATC	Identification of T-DNA insertion in the sr34 mutant
22	SALK_106067C-RP	TCCAAGTGATCCAATGTCTCC	Identification of T-DNA insertion in the sr34 mutant
23	SALK_124563C-LP	ACCGGTGTTTGTAAATTGCAG	Identification of T-DNA insertion in the sc35 mutant
24	SALK_124563C-RP	GGCTTCTTCTGGGACTACGAC	Identification of T-DNA insertion in the sc35 mutant
25	SALK_063528C-LP	TTGAGAGAGAGGTCGAGCTTG	Identification of T-DNA insertion in the sr45 mutant
26	SALK_063528C-RP	CAATTCGAATTCGAGCTGAAC	Identification of T-DNA insertion in the sr45 mutant
27	SALK_024285C-LP	AATACTTGGCCATATCGCTCC	Identification of T-DNA insertion in the cbp80 mutant
28	SALK_024285C-RP	TAATTTAGGCTCTTCCGGGTG	Identification of T-DNA insertion in the cbp80 mutant
29	LBb1.3	ATTTTGCCGATTTCGGAAC	Identification of T-DNA insertion in the mutant
30	circP5CS1-F	GACTTGTGACTTCAAGAGAAGAG	qRT-PCR
31	circP5CS1-R	CAATGAGTTTACCCCCAACAG	qRT-PCR
32	circP5CS1-mut-F	GGTCATGCTGATTGGAAG	qRT-PCR
33	circP5CS1-mut-R	AGTTTACCCCCAACAGTC	qRT-PCR
34	miR159-F	CGGCGGTTTGGATTGAAGGGA	qRT-PCR
35	miR159-RT	GTCGTATCCAGTGCAGGGTCCGAGGTATTCGCA CTGGATACGACTAGAGC	RT
36	miR167-F	CGGCGGTGAAGCTGCCAGCAT	qRT-PCR

37	miR167-RT	GTCGTATCCAGTGCAGGGTCCGAGGTATTCGCA CTGGATACGACCTAGATC	RT
38	Universal-R	GTGCAGGGTCCGAGGT	qRT-PCR
39	U6 -RT	GTGCAGGGTCCGAGGTTTTGGACCATTTCTCGA T	RT
40	U6-F	GGAACGATACAGAGAAGATTAGCA	qRT-PCR
41	ARF6-F	TTGGCGATCTGTCAAGGTTGGG	qRT-PCR
42	ARF6-R	ACCAAGCAAGCCCGACGTAT	qRT-PCR
43	ARF8-F	AGGGTCACAGTGAACAGGTAGC	qRT-PCR
44	ARF8-R	CTGCAGTGTGTAATCCAATGGAGGA	qRT-PCR
45	MYB33-F	TCGTCATCTCCTCCACACTCTG	qRT-PCR
46	MYB33-R	CCTCGGATTTAGTTTGGGATAC	qRT-PCR
47	SE-F	CGTAAACCCAGCATGGAGAACAG	qRT-PCR
48	SE-R	GGAGCATCTAGGTCTTGGTAGC	qRT-PCR
49	RSZ22-F	GCGAGTTACTGAGCGTGAACTTG	qRT-PCR
50	RSZ22-R	GAGATCTTGCACGAGGGCTG	qRT-PCR
51	RS31-F	GCAATTTCGAGTATGAAACTCGC	qRT-PCR
52	RS31-R	GCTTCTTTGAGTAGCTTCAAGGG	qRT-PCR
53	SCL33-F	ATGAGGGGAAGGAGCTACACTC	qRT-PCR
54	SCL33-R	CGGTTTCTTTCTGTTCTCTTCTG	qRT-PCR
55	SR34-F	ATGAGCAGTCGTTCGAGTAGAAC	qRT-PCR
56	SR34-R	CTTGCCAAGAAGCAGATGAAGG	qRT-PCR
57	SC35-F	GTCAGGTCCACCGGATATCAG	qRT-PCR
58	SC35-R	CTTCTTCTGGGACTACGACGTCTAG	qRT-PCR
59	SR45-F	GTGTCTGGCTCGTCATCTCG	qRT-PCR
60	SR45-R	GAGCTGAGCTTTCTCAGCATCAG	qRT-PCR
61	CBP80-F	GGAAAACTCTTCTCCTTCGCATC	qRT-PCR
62	CBP80-R	GTGGCTGCCATGATGGATTTCC	qRT-PCR
63	LOX2-F	CCATTGGTTGAGGACTCATGC	qRT-PCR
64	LOX2-R	CCTCCGTTGACAAGACTTTGG	qRT-PCR
65	AOS-F	GCGGAGGAGATTAGATCTGTG	qRT-PCR
66	AOS-R	GATCCTTCTTCGCTCTACCG	qRT-PCR
67	OPR3-F	GCTTCTCAGCAGTGTATCAAC	qRT-PCR
68	OPR3-R	TATCTCGGAAGCTTCTAAAGCC	qRT-PCR
69	ICS1-F	CGAAAGACGACCTCGAGTTC	qRT-PCR
70	ICS1-R	CTGGAGTTGGATGCAGAGC	qRT-PCR
71	PR1-F	CCACAAGATTATCTAAGGGTTC	qRT-PCR
72	PR1-R	GGCTTCTCGTTCACATAATTCC	qRT-PCR
73	GDG1-F	CTCGTACTCGAGTCATCAGG	qRT-PCR
74	GDG1-R	GCGGATCCCCAAGGAAGAACAATCAAATACTG	qRT-PCR
75	YUC6-F	GGGCTGTCCATGTTCTTACTGAA	qRT-PCR
76	YUC6-R	CGGGAAACAACCAAAAGGAA	qRT-PCR
77	IAA19-F	TCGGTGTGGCCTTGAAAGATGG	qRT-PCR

78	IAA19-R	TGCATGACTCTAGAAACATCCCCC	qRT-PCR
79	circP5CS1-RNAi-F1	GAGTGAAGCTATCAGCATGACCTTCTCTCTTTTGTATTCC	circP5CS1-RNAi construct
80	circP5CS1-RNAi-R2	GAAGGTCATGCTGATAGCTTCACTCAAAGAGAATCAATGA	circP5CS1-RNAi construct
81	circP5CS1-RNAi-F3	GAAGATCATGCTGATTGCTTCACTCACAGGTCGTGATATG	circP5CS1-RNAi construct
82	circP5CS1-RNAi-R4	GAGTGAAGCAATCAGCATGATCTTCTACATATATATTCCT	circP5CS1-RNAi construct
83	circP5CS1-OE-F1	CGTCTAGAGTGAAAACTAAACACGAAAGAACAC	circP5CS1-OE construct
84	circP5CS1-OE-R2	GGGAAATGAATTTTGACTATTAACATACCTGTACAAGTTTGGCAGCATAA	circP5CS1-OE construct
85	circP5CS1-OE-F3	TTATGCTGCCAAACTTGTACAGGTATGTTAATAGTCAAAATTCATTTCCC	circP5CS1-OE construct
86	circP5CS1-OE-R4	GGATGGCAAGTGAAGCTATCTGTTTGGTTTGTGGATAAAGC	circP5CS1-OE construct
87	circP5CS1-OE-F5	GCTTTATCCACAAACCAAACAGATAGCTTCACTTGCCATCC	circP5CS1-OE construct
88	circP5CS1-OE-R6	GAAAAAACAAACTTGCAACCATACCAGCATGACCTAGCACAGG	circP5CS1-OE construct
89	circP5CS1-OE-F7	CCTGTGCTAGGTCATGCTGGTATGGTTGCAAGTTTGTTTTTTC	circP5CS1-OE construct
90	circP5CS1-OE-R8	TTATGCTGCCAAACTTGTACAGCTGCAAGTACACAATTAAGCCA	circP5CS1-OE construct
91	circP5CS1-OE-F9	TGGCTTAATTGTGTACTTGCAGCTGTACAAGTTTGGCAGCATAA	circP5CS1-OE construct
92	circP5CS1-OE-R10	CGCTGCAGGTGAAAACTAAACACGAAAGAACAC	circP5CS1-OE construct
93	circP5CS1-mut-OE-F1	CATTGGACTTGTGACTTCAAGAG	circP5CS1-mut-OE construct
94	circP5CS1-mut-OE-R2	GATGGCAAGTCTTCCAATCAGCATGACCTAGCACAGG	circP5CS1-mut-OE construct
95	circP5CS1-mut-OE-F3	CTAGGTCATGCTGATTGGAAGACTTGCCATCCGTAGTG	circP5CS1-mut-OE construct
96	circP5CS1-mut-OE-R4	AGTTTACCCCCAACAGTCTCTG	circP5CS1-mut-OE construct
97	miR167a/b-OE-F1	GATGAAGCTGCCAGCATGATCTATCTCTCTTTTGTATTCC	miR167a/b-OE construct
98	miR167a/b-OE-R2	GATAGATCATGCTGGCAGCTTCATCAAAGAGAATCAATGA	miR167a/b-OE construct
99	miR167a/b-OE-F3	GATAAATCATGCTGGGAGCTTCTTCACAGGTCGTGATATG	miR167a/b-OE construct
100	miR167a/b-OE-R4	GAAGAAGCTCCCAGCATGATTTATCTACATATATATTCCT	miR167a/b-OE construct
101	pRS300-A-F	CTGCAAGGCGATTAAGTTGGGTAAC	circP5CS1-RNAi/miR167ab-OE construct
102	pRS300-B-R	GCGGATAACAATTTCACACAGGAAACAG	circP5CS1-RNAi/miR167ab-OE construct

103	STTM-common-real-F	CATTTGGAGAGGACAGCCCAAG	miR167-STTM construct
104	STTM-common-real-R	CTGGTGATTTCAGCGTACCGAA	miR167-STTM construct
105	PacI-del-F	TCCCTTAATTAAGTTTGCAAGCAGCAGATTACGCG	miR167-STTM construct
106	PacI-del-R	TCCCTTAATTAAGAAAGGCGGACAGGTATCCGGTAAG	miR167-STTM construct
107	STTM167-F	GCCATTTAAATATGGTCTAAAGAAGAAGAATTAGATCATGCTCAAGGCAGCTTCAGAATTCGGTACGCTGAAATCACCAG	miR167-STTM construct
108	STTM167-R	GCCATTTAAATTAGACCATAACAACAACAACTGAAGCTGCCTTGAGCATGATCTAAAGCTTGGGCTGTCCTCTCCAAATG	miR167-STTM construct
109	5-BIO-miR167a/b	UGAAGCUGCCAGCAUGAUCUA	Probe for miRNA pull down
110	5-BIO-miR167c	UAAGCUGCCAGCAUGAUCUUG	Probe for miRNA pull down
111	5-BIO-miR167d	UGAAGCUGCCAGCAUGAUCUGG	Probe for miRNA pull down
112	5-BIO-miR159a	UUUGGAUUGAAGGGAGCUCUA	Probe for miRNA pull down
